# The Evolutionary Fate of Phenotypic Plasticity and Functional Traits under Domestication in Manioc: Changes in Stem Biomechanics and the Appearance of Stem Brittleness

**DOI:** 10.1371/journal.pone.0074727

**Published:** 2013-09-04

**Authors:** Léa Ménard, Doyle McKey, Gilda S. Mühlen, Bruno Clair, Nick P. Rowe

**Affiliations:** 1 Université Montpellier 2, UMR AMAP, Montpellier, France; CNRS, UMR AMAP, Montpellier, France; 2 Université Montpellier 2, Centre d’Ecologie Fonctionnelle et Evolutive (CEFE), UMR 5175 CNRS, Montpellier, France; 3 Institut Universitaire de France, Paris, France; 4 Department of Agronomy, Federal University of Rondônia, Rolim de Moura, Rondônia, Brazil; 5 CNRS, Laboratoire de Mécanique et Génie Civil (LMGC), Université Montpellier 2, Montpellier, France; 6 CNRS, UMR Ecologie des Forêts de Guyane (EcoFoG), Campus Agronomique, Kourou, French Guiana; New York State Museum, United States of America

## Abstract

Domestication can influence many functional traits in plants, from overall life-history and growth form to wood density and cell wall ultrastructure. Such changes can increase fitness of the domesticate in agricultural environments but may negatively affect survival in the wild. We studied effects of domestication on stem biomechanics in manioc by comparing domesticated and ancestral wild taxa from two different regions of greater Amazonia. We compared mechanical properties, tissue organisation and wood characteristics including microfibril angles in both wild and domesticated plants, each growing in two different habitats (forest or savannah) and varying in growth form (shrub or liana). Wild taxa grew as shrubs in open savannah but as lianas in overgrown and forested habitats. Growth form plasticity was retained in domesticated manioc. However, stems of the domesticate showed brittle failure. Wild plants differed in mechanical architecture between shrub and liana phenotypes, a difference that diminished between shrubs and lianas of the domesticate. Stems of wild plants were generally stiffer, failed at higher bending stresses and were less prone to brittle fracture compared with shrub and liana phenotypes of the domesticate. Biomechanical differences between stems of wild and domesticated plants were mainly due to changes in wood density and cellulose microfibril angle rather than changes in secondary growth or tissue geometry. Domestication did not significantly modify “large-scale” trait development or growth form plasticity, since both wild and domesticated manioc can develop as shrubs or lianas. However, “finer-scale” developmental traits crucial to mechanical stability and thus ecological success of the plant were significantly modified. This profoundly influenced the likelihood of brittle failure, particularly in long climbing stems, thereby also influencing the survival of the domesticate in natural situations vulnerable to mechanical perturbation. We discuss the different selective pressures that could explain evolutionary modifications of stem biomechanical properties under domestication in manioc.

## Introduction

### Domestication and growth forms

The term “domestication syndrome” refers to the suite of traits that evolve under domestication and that have adapted plants to agricultural environments. Some traits known to be modified by domestication include increased yield, improved nutritional and organoleptic qualities and facilitated propagation. Under domestication, traits can evolve via intentional or unintentional human selection or by natural selection processes [1], [2].

Plant growth form and architecture have frequently undergone evolutionary change under domestication. In cereals, domestication usually results in a reduction in the degree of branching, adapting the crop to growing at high densities [3]–[5]. Maize, for example, possesses a single little-branched main stem, while its wild relative teosinte has a much greater degree of axillary branching, resulting in a bushier organisation. This change under domestication has a genetic base [4]; teosinte usually grows in open conditions and occupies space by branching, whereas maize is usually planted at high densities where its single main stem is adapted for dense growth in agricultural environments. Architecture of teosinte and maize differs in a further important aspect, its plasticity. When teosinte grows in open situations, it branches much more than when grown with surrounding plants. In contrast, when maize is grown without surrounding plants, it is incapable of increasing its degree of branching [6]. Domestication thus appears to have resulted in a reduced plasticity in growth form in response to environmental variation.

Domestication has also led to reduced branching in many other domesticates, including millet [7], rice [8] and bean [9]. Other significant architectural changes have also been produced during domestication.

In this study, we focus on how growth form and stem development have evolved under domestication in the vegetatively propagated woody crop, manioc. This is of particular interest, for three reasons. First, domestication of manioc involved a dramatic change in habitat in comparison to the wild ancestor and this should have led to very different selection pressures on growth form and architecture between wild and domesticate. Second, during domestication the stems of manioc acquired a new function, that of providing clonal propagules (stem cuttings). Selection driven by this new function may have produced new adaptations, and these may be expected to affect the development and structural properties of stems. Third, study of domestication syndromes has been generally neglected in vegetatively propagated crops, partly because of the widespread view that their domestication was a simple process, namely, “instant domestication” by the capture and clonal multiplication of mutant wild genotypes [10], [11]. By the scenario of “instant domestication”, it is difficult to envisage how plants could accumulate different, independently evolved adaptations to agriculture, i.e., how they could have evolved a “domestication syndrome”. In landrace populations of manioc, however, it has been shown that farmers regularly incorporate as new clones, plants issued from volunteer seedlings [10], [11]. The resulting mixed clonal/sexual reproductive system means that there is scope for repeated recombination/selection cycles, as in seed-propagated crops, which would allow the accumulation of adaptations to agriculture.

Biomechanical properties of stems are affected by many developmental traits at varying levels of complexity and probably by many genes. We wished to examine the magnitude and diversity of evolutionary modifications in traits of manioc stems under domestication.

### Plant biomechanics

Comparative studies of growth forms and the biomechanical organisation underlying different growth forms can help us interpret the ecological significance of stem developmental traits and the selection pressures acting on them [12], [13]. Such studies are of particular interest when different species vary in ecology or habitat preference, developing as different growth forms such as herbs, shrubs, trees or climbers. Furthermore, species may vary their organisation and growth form according to different habitats, showing high levels of growth form plasticity and modifying their structural organisation during ontogeny to meet changing environmental and/or mechanical constraints. Such changes during development can lead to variations that strongly affect biomechanical properties of stems [12], [14]–[21].

Many types of climber have evolved various mechanical strategies where dependence on external mechanical supports and economising on structural materials result in narrow, compliant stems [22]. Since slender stems cannot withstand extreme mechanical forces unless they develop specialized mechanical properties, many vines (non woody) and lianas (woody) produce highly flexible stems. These are often capable of resisting high mechanical stresses in shear, bending, torsion and tension and limiting catastrophic failure and damaging fracture of the entire stem. Structural and mechanical differences between shrubs and lianas are of particular relevance for understanding how plants such as species of *Manihot* can develop diverse shrub-like or liana-like phenotypes according to agricultural/natural or open/ closed environmental conditions [23]. Finally, plant stems are strongly hierarchical structures and their material properties result from developmental traits at several scales. These traits range from the overall relative volumes and positions of the main tissues such as pith, wood cylinder and surrounding cortex, to finer-scale developmental features including wood and fibre density and stiffness, down to ultrastructural organisation, including cell wall organisation and cellulose microfibril angle.

### Domesticated manioc

Under cultivation, domesticated manioc (*Manihot esculenta* subsp. *esculenta* Crantz, Euphorbiaceae) is a woody shrub reaching up to four metres in height [24], [25]. It is cultivated for its tuberous roots, which are rich in starch. Manioc was first domesticated in South America [26], but is now planted throughout the tropics and is estimated to be the second most harvested crop in the least developed countries and the fourth most harvested starch crop in the world [11], [27]. The plant is prized for its drought-resistance and for its ability to produce viable yields on nutrient-poor, acid soils where no other crops will grow [28].

Landrace populations of manioc cultivated by Amerindians in Amazonia, the crop’s region of origin, are our best contemporary analogues of the kinds of environments in which manioc has been grown throughout most of its history. The plant is usually grown in long-fallow swidden systems (slash-and-burn agriculture). After a fallow ranging in length from a few years up to a few decades, secondary vegetation is slashed and burned during the dry season, and the crop is planted in new fields at the beginning of the rainy season. The plant is propagated by stem cuttings (‘stakes’). Stakes are cut from mature stems of moderate to large diameter (usually at least 15–20 mm diameter, although exceptionally as small as 8 mm) and of varying length (usually at least 30 cm) [29]. Stakes are planted in the ground, usually at an angle, with about half of the stake protruding from the soil, but cultivators sometimes completely bury them, more or less horizontally, in the soil. Adventitious roots are produced at the cut end of the stake and from meristematic areas either side of the leaf scar at nodes.

Plants of domesticated manioc grow as shrubs, usually free-standing, but occasionally, when planted at high densities, plants lean on neighbouring individuals. Degree of branching varies greatly among landraces [29]–[31].Time to harvest, and thus also the size of the plant, also vary greatly among landraces, some typically being harvested after periods as short as three to six months, others remaining in the ground for up to two years. At harvest, plants are usually uprooted, although plants are occasionally abandoned in fallows. In newly cleared fields, we have never seen plants that could have originated from such abandoned individuals that survived over the entire fallow period. Volunteer seedlings, however, are often abundant in new fields, having lain dormant during the fallow and germinating in response to the disturbance of field clearing and burning [32]. Amerindian farmers allow volunteer seedlings to grow, and some are used by farmers to prepare stem cuttings, adding new clones to landraces. These products of sexual reproduction are important in the evolutionary dynamics of this clonally propagated crop [28], [32]–[39]. Notably, it is their incorporation that leads to the recombination/selection cycles necessary for the accumulation of numerous adaptations during domestication.

There exists an enormous diversity of landraces of manioc [40], varying in numerous traits. We studied growth form and mechanical architecture of several landraces of domesticated manioc in two widely separated parts of the crop’s range in the continent of origin, French Guiana and the southern Brazilian state of Rondônia. Our sample of domesticated manioc is thus broad and, we believe, representative.

### Manioc’s wild ancestors

Recent molecular analyses indicate that the closest wild relative of manioc is *M. esculenta* subsp. *flabellifolia*, and that manioc was domesticated only once, from populations of this taxon growing along the southwestern rim of Amazonia [41]–[45]. Species limits of manioc are unclear. Allem [41] placed several named species in synonymy with *M. esculenta* subsp. *flabellifolia*, including *M. tristis* and *M. surinamensis* found in the Guianas. More recent and inclusive phylogenies of the genus indicate that the genus *Manihot* diversified secondarily in South America and confirm the close relationship of these and a few other described taxa to domesticated manioc [45].

Following this view, a single, highly variable taxon, *M. esculenta* subsp. *flabellifolia*, ranges all along the seasonally dry southern, eastern and northern margins of the Amazon Basin. Certainly its northern members, i.e., those furthest from the region of domestication and most distant genetically from domesticated manioc, still hybridize readily with manioc [46]. In a previous study, seedling functional morphology was found to be very similar in wild populations from Rondônia and the Guianas, and both differed in these traits from domesticated populations [47]. Note: in reference [47], the wild taxon in French Guiana was mistakenly identified as *M. pruinosa* Pohl, another species closely related to domesticated manioc - see reference [45].

Throughout its range, this taxon typically grows in disturbed forest-savannah ecotone habitats. In southwestern Brazil (Rondônia), *M. esculenta* subsp. *flabellifolia* is found as a shrub along roadsides and (more commonly) as scrambling or lianescent plants in remnant patches of low-stature secondary forest. In French Guiana (where it has usually been known as *M. surinamensis* or *M. tristis*), plants usually grow in more open vegetation, as shrubs in savannah or as scrambling, occasionally lianescent, plants in low-stature secondary woodland vegetation. For simplicity, in this study we refer to wild taxa from both Brazil and French Guiana simply as the “wild taxon”. For domesticated manioc *Manihot esculenta* subsp. *esculenta*, we refer simply to the “domesticated taxon”.

The domestication of manioc began as early as 8000–10000 years ago [48]. Little is known about how the earliest farmers of the crop may have manipulated the plant in cultivation. A recent study proposes that manioc is a classical dump-heap crop [49]: stems of harvested wild manioc plants, discarded in dump heaps after removal of the roots, sprouted and grew well in these nutrient-rich environments. Noticing this, people began planting stem segments in these sites. Little is known of the toolkit these incipient farmers may have had available, but it certainly included digging sticks and stone axes. Wooden or stone knives are plausible possibilities, but there is no archaeological or ethnographic record suggesting wooden knives may have been used by the earliest cultivators of manioc, and stone knives were probably few in number (Charles Clement, INPA Manaus, pers. comm.).

We compared stems of manioc and those of its wild relatives from both Rondônia and French Guiana; both are pertinent to the study of evolution under domestication. Wild populations from Rondônia, the area of origin, are genetically most closely related to manioc [26], [44]. These may well be the closest contemporary analogues, in ecological and morphological terms, of manioc’s wild ancestors. However, climate has become wetter in this part of South America during the Holocene [50], and it cannot be excluded that when manioc was domesticated, perhaps 8000 yr. or more in the past [48], its wild ancestors in this region were adapted to drier, more open vegetation types, and resembled in their life form and habitat contemporary populations in areas such as the savannahs of coastal French Guiana. Studying wild relatives from both Rondônia and French Guiana provides us with information on the full range of variation in growth form and ecological conditions likely to have characterized manioc’s wild ancestors at the time of domestication. If traits affecting mechanical architecture are found to be common to wild populations from both areas and to differ from those of the domesticate—as found for seedling functional morphology [47]—this would increase the confidence we can place on our conclusions about evolution under domestication.

We compared stem biomechanics in manioc and its wild ancestors, in order to quantify how the mechanical architecture was modified during the domestication process. We then aimed to assess the morphological and anatomical modifications underlying differences in growth habit. We addressed the following questions based on these two approaches:

(1) Do manioc and its wild relatives develop the same or different growth forms depending on the environmental conditions encountered by the plant (or by different parts of the plant’s shoot system)?

(2) Are there differences in stem mechanical properties between wild and domesticated taxa and is the developmental plasticity of these traits maintained in the domesticated taxon?

(3) If mechanical architecture is modified by domestication, what selective pressures were responsible? Did mechanical traits change as a consequence of selection on other traits? For example, if domestication of manioc involved a shift from resource-conservation to resource-acquisition strategies, as is frequent in domesticated plants, this could affect the composition, and thereby the biomechanical traits, of manioc stems. Or did selection act directly on mechanical properties? Whereas farmers today prepare vegetative propagules by cutting stems into segments, the metal tools used to do this only became available 500 years ago. Stone or wooden tools may have been less efficient, and farmers may have selectively propagated plants whose stems broke easily and cleanly.

## Materials and Methods

### Ethics statement

All necessary permits were obtained for the described study, which complied with all relevant regulations. We thank the National Council of Technological and Scientific Development (CNPq) of Brazil for granting research permits to N. Rowe, L. Menard and D. McKey (Portaria No. 630, 25 September 2007, Proceso EXC 038/06C). For sampling carried out on private land in French Guiana, permission was obtained from the landowners.

Data were collected during two field trips in French Guiana in February and April/May 2007, totalling 40 days of field work, and during a single field trip to Rondônia in August 2008 (7 days of field work). In both areas, we studied domesticated manioc and its wild relatives, including self-supporting and climbing plants of each taxon. A plant was defined as self-supporting if it was upright and stable and contacted no robust vegetation that provided mechanical support. Climbing plants were those that leaned upon, or climbed within, vegetation that surrounded the plant and gave it mechanical support.

### Sampling– French Guiana

#### a) Wild relative (“*Manihot tristis*”)

**Figure 1:**
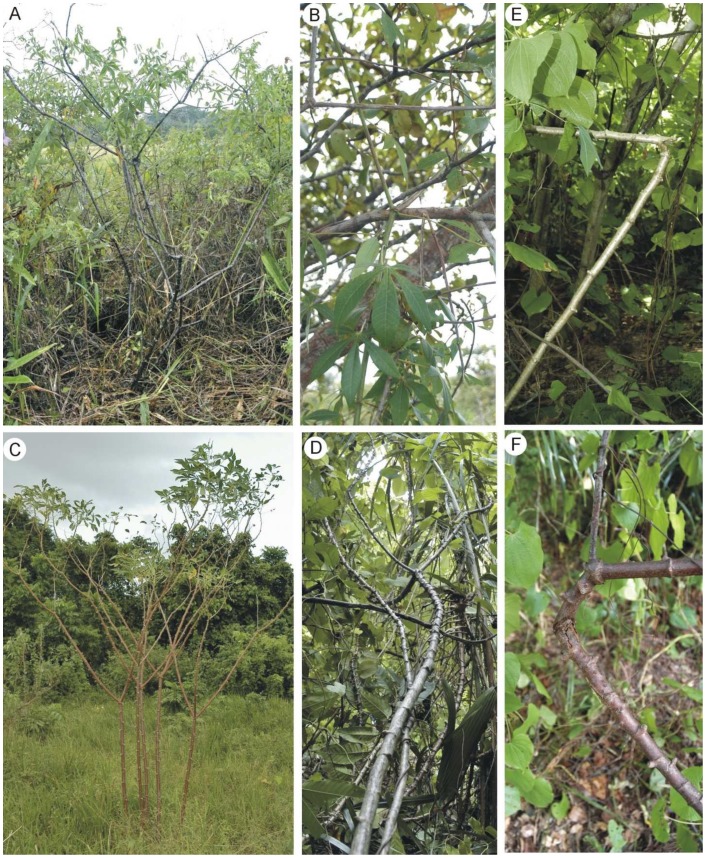
Growth forms of wild and domesticated *Manihot* in French Guiana. (A) Self-supporting shrub of the wild relative (locally known as “*Manihot tristis*”, which is considered synonymous with *M. esculenta* subsp. *flabellifolia* [41]) growing in disturbed savannah, Macouria, French Guiana. (B) Climbing stems of this wild relative in forest margin adjacent to savannah, Macouria, French Guiana. (C) Self-supporting shrubs of domesticated manioc, *Manihot esculenta* subsp. *esculenta*, growing in open conditions on plantation, village of Kamuyene, near Macouria, French Guiana. (D) Climbing stems of domesticated manioc growing in abandoned plantation, village of Kamuyene, near Macouria, French Guiana. (E-F) Brittle fracture in natural growth conditions of climbing stems of domesticated manioc growing in abandoned plantation, village of Kamuyene, near Macouria, French Guiana.

Individuals of the wild relative were harvested in a savannah (Savane Maillard, 4°58’55’’N, 52°27’00’’W) near the village of Macouria where the vegetation is comprised of a mosaic of low-stature secondary forest in a matrix of grasses, dicot herbs and forbs. We sampled in an area where the population included self-supporting individuals in the grassy matrix (Fig. 1 A) and climbing individuals at edges of forest patches and within them (Fig. 1 B). A total of 22 self-supporting individuals and 17 climbing individuals were collected and biomechanical measures performed on them.

#### b) Domesticated manioc (*Manihot esculenta* subsp. *esculenta*)

We sampled domesticated manioc from among landraces (all bitter) grown in the Palikur Amerindian village of Kamuyene (4°59’24’’N, 52°26’43’’W), near the village of Macouria. Self-supporting individuals (Fig. 1C) were collected in active fields, sometimes at the edges in order to sample a range of developmental stages. Manioc plants had been planted with sufficient space around each individual so that mature plants were always self-supporting. We collected and measured 19 self-supporting plants.

Manioc is usually uprooted when the crop is harvested and the plot allowed to go to fallow. However, because of declining yields, three years before our study the Palikur at Kamuyene had moved most of their farming activity to a newly opened area 55 km inland from the village, abandoning many of their manioc farms unharvested. We thus had access to many three-year-old fallows in which vegetation was dense, including palms, young trees and many climbing plants. We found large numbers of abandoned manioc plants that had produced long stems growing into the surrounding vegetation and which had developed climbing phenotypes (Fig. 1D). We collected and measured 15 of these climbing individuals.

### Sampling–Rondônia

#### a) Wild relative (Manihot esculenta subsp. flabellifolia)

**Figure 2:**
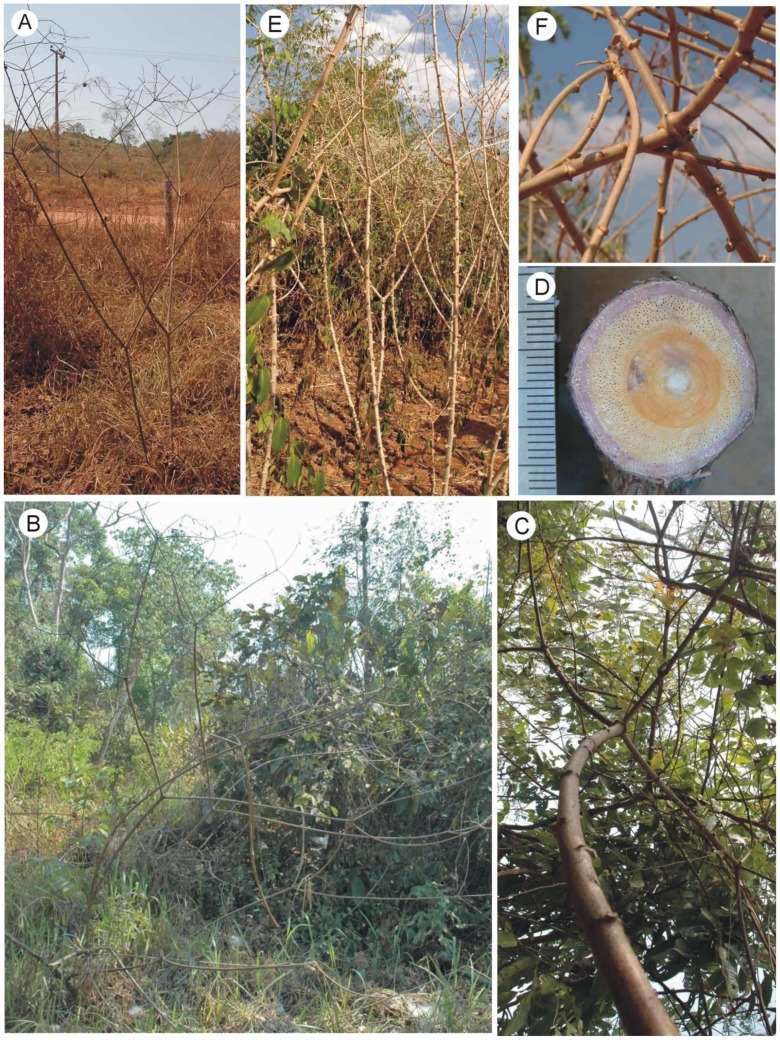
Growth forms of wild and domesticated *Manihot* in Rolim de Moura, Rondônia, Brazil. (A) Self-supporting shrub of the wild taxon *Manihot esculenta* subsp. *flabellifolia* growing in disturbed savannah. (B) Climbing stems of *Manihot esculenta* subsp. *flabellifolia* growing in disturbed forest margin. (C) Climbing stem of *Manihot esculenta* subsp. *flabellifolia* with characteristic mode of climbing for the genus via wide-angled branches. (D) Transverse section of base of climbing stem of *Manihot esculenta* subsp. *flabellifolia*, with an inner zone of dense, stiff wood, followed by a second type of flexible wood with densely distributed vessels. (E) Self-supporting shrubs of domesticated manioc, *Manihot esculenta* subsp. *esculenta*, growing in open conditions. (F) Stand of densely planted shrubs of domesticated manioc, approaching instability and climbing habit with (E) evidence of interlocking, wide-angled branches.

We sampled self-supporting (Fig 2A) and climbing individuals (Fig 2B, C) from populations of this taxon in the vicinity of the town of Rolim de Moura (11°45’38’’S, 61°46’41’’W). Only a short time was available for field work. Due to factors beyond our control we could only schedule this trip during the dry season, limiting the number of plants we could find that were suitable for biomechanical measurements. Only plants in which the entire set of stem axes was vigorous and bearing numerous leaves, were included in our study. These were found in an area that had received exceptional rainfall in July. We collected and measured mechanical properties of five self-supporting individuals (found in herbaceous roadside vegetation) and two extensively branched climbing individuals, that nevertheless yielded 30 measurements of small to large diameter axes (found in remnant secondary-forest patches). Biomechanical measurements – if they are to represent values typical of the living plant - must be carried out on fresh green material. This can be an issue with wild species of *Manihot* since shrub-like growth forms can dry and die back during severe dry seasons. We therefore present results from a limited data set based on fresh green material, which we believe nevertheless is representative of some of the main attributes of the shrubs and climbers we encountered.

#### b) Domesticated manioc (*Manihot esculenta* subsp. *esculenta*)

We collected and measured six individuals, five of them from a single landrace in a small home garden near Rolim de Moura (11°34’58’’S, 61°46’24’’W), and one individual from a different landrace collected from a garden in the town. The owners did not know the names of the landraces, but both were sweet. All plants were self-supporting (Fig. 2 E), but one individual in the home garden had developed several climbing stems. Manioc was planted in exceptionally high density in this garden, and a number of other stems tended to be unstable or climbing via interlocking branches (Fig. 2 F).

### Description, mapping and measuring of plants

Since we suspected that growth forms of manioc change with the environment, we hypothesized that mechanical properties would vary as a function of the plant’s environment and the growth form it adopted. We chose for study mature plants with well-developed branch systems, in open environments (herbaceous savannah or roadside vegetation for wild relatives and active fields for domesticated manioc). We also selected mature plants in closed environments (forest or bush patches for the wild relatives and abandoned fallows for domesticated manioc). Before collection, plants were photographed, neighbouring vegetation being first removed to show clearly the plant’s overall shoot system. A schematic map was then made of the hierarchical set of connected axes that were chosen for biomechanical measurements.

Measurements were made on segments cut from along entire axes and branch systems, in order to follow the variations of mechanical properties and underlying morphological variation. Since climbing stems were often much longer than self-supporting stems, a greater number of measures was taken from them, despite the fact that the number of individual plants measured was similar for the two categories.

#### a) Mechanical tests

Mechanical tests were performed on freshly harvested plants that were kept in humid conditions before testing, always within 48 h following collection. Segments of axes chosen for measurement were straight and with less than 10% tapering along their length. Measurements were made along a set of connected axes and thus represent different stages in the development of the plant. Mechanical properties of stems of manioc and of its wild relatives were quantified via two kinds of tests.

#### b) Elastic properties

We studied bending mechanical properties following recent protocols [15], [18], [22], [23], [51] using an electronic mechanical testing device (In-Spec 2200, Instron Corporation, Norwood, MA, USA; http://WWW Instron.Com). The machine consists of a motorized cross-head linked to a load cell that measures force applied and vertical displacement. Some measurements were also carried out on a manually operated bending apparatus [22]. Span tests were performed to establish the minimum span-to-depth ratio at which the effect of shear could be neglected [51]. For all stages of development of both wild and domesticated *Manihot* taken together, a span-to-depth ratio of 25 was necessary, similar to that found for *Manihot quinquepartita* [23]. Material from French Guiana was studied using a manual method, whereby weights are placed on a pannier suspended from the middle of the stem [22]. For each measurement, five weights were added in succession, and vertical deflexion of the stem (considered to have reached its maximum after 30 sec) was measured using a binocular microscope after addition of each weight. Weights were chosen so as to produce small incremental increases in deflexion, thus remaining within the elastic range of the material. A total of 398 such measurements were performed on stems of manioc and its wild relative in French Guiana. The material from Rondônia was studied using the portable Instron apparatus.

A total of 59 3-point bending tests were conducted on stems of manioc and its wild relative in Rondônia.

The Young’s elastic modulus in bending (*E*), expressed in MPa, was derived from the following formula
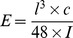
(1)


where *I* is the axial second moment of area (mm^4^) of the tested segment. This parameter quantifies the quantity of material within a cross-section and its distance from the neutral axis in bending through the integral of surface elements multiplied by the distance squared to the neutral axis. For an ellipse, it corresponds to the following formula:
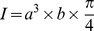
(2)


Where *a* is the radius of the tested stem in the direction of the applied force and *b* the radius perpendicular to the applied force.

When measured in bending, the Young’s modulus quantifies the capacity of a material to resist bending. This parameter is independent of the geometry and the size of the tested sample [17], [52]. It corresponds to the ratio between applied stress and resulting strain and is conveniently calculated by dividing the flexural rigidity by the second moment of area. Strictly speaking, the term “Young’s modulus” is usually applied only to isotropic materials. For heterogeneous biological materials such as plant stems, the term “structural Young’s modulus” is more appropriate [17]; for simplicity, we use the term “Young’s modulus” here.

#### c) Failure properties

Failure tests were carried out using the motorized Instron apparatus in both Rondônia and French Guiana. We followed general protocols applied elsewhere on biological materials [53] including plants [54]. In these tests the load is applied to the specimen well beyond the elastic limit until collapse of the stem. The tested specimen is positioned on two supports with the force applied at the centre point. In all rupture tests the force was applied at a constant speed of 0.25 mm.s^−1^. The span between the two supports was adjusted depending on the stem diameter to a span-to-depth ratio of 15 for all tested stems, ensuring that failure occurred for most stems tested while remaining within the limit of 500 N for the machine. Loads were applied using one of several cylindrical metal probes attached to the cross-head. In order to control a potential “knife edge” effect where the descending probe might cut a notch in the tested stem, a probe diameter was chosen for each tested segment that was no smaller than the diameter of each stem segment under test.

3-point bending is not ideal for strictly quantifying bending stresses leading up to failure for at least two reasons. First, the stresses occurring within the stem with this kind of set-up do not only involve stresses in bending since with a span-to-depth ratio of 15, shear forces are likely operating at small deformations and significantly at large deformations. Second, unlike failure tests in bending with a 4-point set-up, the force is applied at a single point of the stem and at high loads is more likely to develop an indentation that could concentrate stresses and initiate failure via the development of a notch. Despite these drawbacks, a series of initial tests suggested that 3-point bending tests to failure with a constant span-to-depth ratio and controlling the diameter of the cross head probe, did offer a means of comparing failure characteristics between the different taxa and growth forms of *Manihot*.

We compared two characteristics from the failure tests. First, the maximum bending stress σ_max_, given by the formula
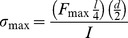
(3)


where *F*_max_ is the force applied (N), *l* is the span length (mm) between the two supports of the 3-point bending test, *d* is the vertical diameter (mm) of the stem at the centre of the test and *I* is the axial second moment of area (mm^4^) of the plant stem approximated as an ellipse in cross-section (see above).

Second, we categorized all stress/strain curves into four generalized kinds of failure based on the form of the curve following yield. We considered this as a suitable semi-quantitative measure of stem brittleness with post-yield deformations and failures ranging from (a) smooth, (b) notched, (c) stepped to (d) rapid total reduction in stress values.

A total of 232 failure tests were made on material from French Guiana (121 domesticated, 211 wild) and 61 tests were made on material from Rondônia (16 domesticated, 45 wild).

#### d) Internal tissue geometry and anatomy

Following mechanical tests, stem segments were preserved in ethanol (60%) for anatomical study; segments were trimmed adjacent to the applied force and the cut surface polished with fine-grain emery paper under running water. Contours of main tissues (outer cortex/bark, wood and pith) were traced and digitized. Surface areas and second moments of areas of each tissue were then calculated via a macro in the commercial image analysis programme “Optimas”, V.6.5.172, Media Cybernetics, Inc., Rockville, MD, USA. Measurements of cell lumen diameter and cell wall thickness of wood fibres were taken from the basal-most segment of each tested plant. Two hand-cut sections were prepared from each cross-section from opposite halves of the wood cylinder. Sections were stained in safranin. Fibre cell lumen size was measured from the major and minor axes of the fibre cell lumen (considered an ellipse) and the double wall thickness from four, evenly spaced points around the fibre cell. A total of ten cells was measured in each opposite hand-cut section, each from a microscope field of 220×166 µm—a total of 20 cells measured per segment cross-section. Vessel lumen density (area µm^2^/mm^2^) was measured from contours traced around each vessel lumen using the software “Image J” from the two opposite sections, each from a microscope field of 2.19×1.65 mm.

#### e) Microfibril angle

We investigated two ways in which microfibril angle (MFA) varied during development in both wild and domesticated shrubs and climbers: (1) how MFA varied from the inside to the outside of the wood cylinder in the basal part of the stem *(c.* 0.3 to 0.5 m above ground level) and (2) how microfibril angle of the outermost part of the wood cylinder varied with elastic modulus (measured in bending).

Microfibril angles (MFA) were measured on three specimens of each phenotype (shrub and climber) of each taxon (wild and domesticated), i.e., a total of 12 plants. Wood samples were cut with a fresh razor from the wood cylinder and cut into strips 0.5×2.0×20 mm (radial, tangential and longitudinal, respectively).

For variations along the radial width of the wood cylinder, we extracted wood samples from two to four positions (depending on the stem diameter) from the pith (earlier-formed tissue) to the bark (later-formed) representing inner, inner middle, outer middle and outer parts; care was taken to avoid zones of tension wood (dark crescents in transverse section), which can generate unrepresentative low values [55]

For tracing variations with Young’s modulus, three segments from the lower part of each plant were selected (0.3 to 2.5 m above ground level for shrubs and 0.3 to 4 m for climbers). Only the MFA measurement taken from the peripheral part of the wood cylinder was plotted against Young’s modulus, as mechanical properties of a stem are mainly affected by the material at the periphery of the cylinder.

Measurements were carried out using X-ray diffraction (XRD). Analyses used a 4-circle diffractometer (Gemini, Agilent Technologies, Santa Clara, USA) equipped with a CCD camera (1024×1024 pixels). CuKα radiation was produced by an X-ray generator operating at 50 kV, 25 mA. Images were integrated between 2θ = 21.5 and 23.5 along the entire 360° azimuthal interval in order to plot the intensity diagram of the (200) plane (for notation of plane orientations in solid state physics see [56]. An automatic procedure allowed the detection of peaks for the (200) plane and their inflexion points. The T parameter [57] was measured as the half distance between intersections of tangents at inflexion points with the baseline. The average MFA of each sample was estimated using the “improved Cave’s method” [58]. The results are given as the means of values obtained for the two peaks for the (200) plane.

Statistical analyses were conducted using the commercial software “Statistica”, StatSoft, Inc. Tulsa, OK, USA. Comparisons of elastic properties, maximum bending stress and xylem tissue traits were carried out using non-parametric Mann-Whitney tests. Differences in failure modes were explored using chi^2^ tests and the potential influence of wood cylinder geometry on stem stiffness was investigated using non parametric Spearman rank correlation coefficients (R_s_).

## Results

### Growth form and habitat

#### The wild relatives

In French Guiana, growth forms of the wild relative were closely linked to the environment. In open areas dominated by herbaceous vegetation, *M. tristis* only grew as self-supporting shrubs up to 3 m high with stem diameters up to 20 mm (Fig. 1 A) and did not develop procumbent, scrambling or climbing forms. In overgrown brush, wooded areas and forest margins, the species developed climbing growth forms with stems up to 8 m in length that attached via woody stipules, leaf petioles and wide-angled branches (Fig. 1 B). This taxon flowers and produces fruit as shrubs. Young plants in closed vegetation, not yet reaching surrounding vegetation, differed from mature individuals in always developing a self-supporting growth form (chi^2^ test, d. f. = 1, p<0.001).

In Rondônia, growth forms of *M. esculenta* subsp. *flabellifolia* were also linked to the environment. Self-supporting individuals were characteristic of open herbaceous roadside vegetation (Fig. 2 A), whereas individuals in wooded vegetation were scrambling to lianescent, depending on the stature of the surrounding vegetation (Fig. 2 B, C). Viewed macroscopically, cross-sections of climbing stems showed a characteristic wood development typical of other wild species of *Manihot* [23] and many other lianas [22] with distinct inner and outer zones of wood (Fig. 2, D). Wild relatives in both Brazil and French Guiana showed very similar variations of growth form with the environment and density of the vegetation. The Brazilian plants, however, tended to be larger than in French Guiana, with basal stem diameters of up to 50 mm. Brazilian plants also developed (a) adventitious roots in contact with the soil, (b) thicker young shoots and (c) larger leaves than those in French Guiana. Our observations also suggested a further difference, whereby Rondônia populations attained greatest fruit production as scrambling or lianoid plants, whereas those in French Guiana attained greatest fecundity as large self-supporting individuals. This suggests a modal (but not absolute) difference between wild relatives in these two regions considered at mature stages of growth.

#### b) Domesticated manioc

In French Guiana, domesticated manioc plants were self-supporting in all actively cultivated plots. These corresponded to the classic description of the plant, usually cultivated as a shrub in Amazonia, in monospecific plantations. In cases where manioc plantations had been abandoned, such as the village of Kamuyene in coastal French Guiana and the village of Saül in central French Guiana, individuals developed climbing growth forms in the encroaching forest (Fig. 1, D). Abandoned plots found in Kamuyene were colonized by palms, small trees and vines, forming an environment that was quite different from actively cultivated plots in terms of luminosity and intensity of competition. Manioc plants in these abandoned plots responded by producing long stems that climbed over the surrounding vegetation or crept on the ground. These phenotypes were attached to surrounding vegetation in the same way as observed for wild species of *Manihot* via wide-angled branches. In contrast to climbing stems of wild relatives, many climbing or trailing stems of domesticated manioc showed evidence of brittle failure (Figs. 1 E, F) where woody stems showed complete transverse fractures. Observations suggested that these resulted from dislodging from surrounding vegetation and subsequent buckling, rather than more active disturbance such as that produced by a falling tree or branch.

In Rondônia, plants grew as self-supporting shrubs under cultivation (Fig. 2, E). Stands of the domesticate included relatively aged individuals that appeared to approach a climbing habit, by leaning against neighbouring individuals and interlocking wide-angled branches with them (Fig. 2 E, F).

### Mechanical properties of the stem

#### Elastic bending properties of the stem (Young’s modulus)

**Figure 3:**
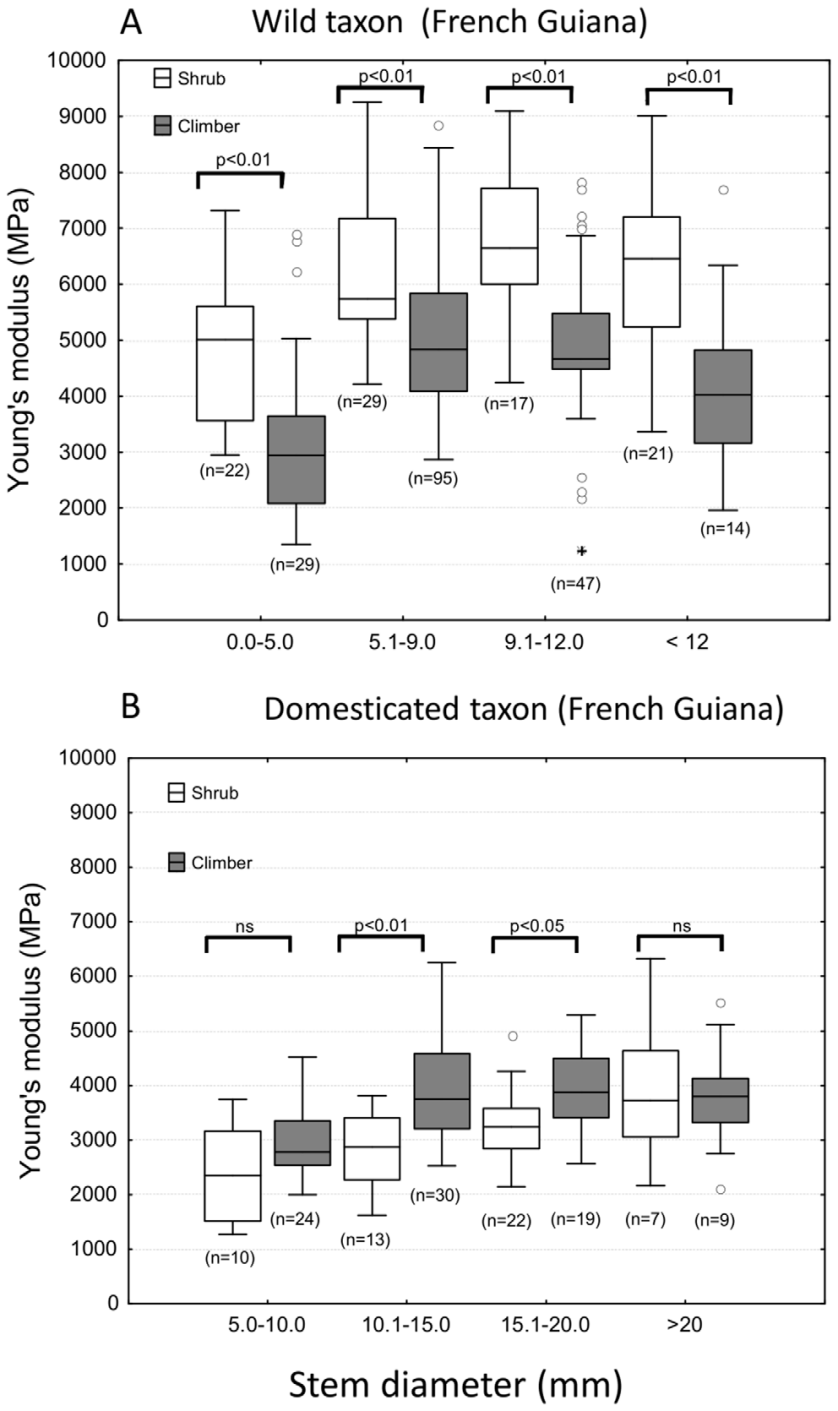
Box plots of Young’s modulus for shrub and climber phenotypes of wild and domesticated manioc. (A) Shrubs of the wild relative from French Guiana have stems comprised of consistently stiffer materials than climbing stems in all size classes. (B) Stems of domesticated *M. esculenta* from French Guiana are generally less stiff than those of the wild taxon and there is a less consistent difference in stiffness between shrubs and climbers in the domesticate. Sample sizes (box plots: medians, 1^st^, 3^rd^ interquartiles, outliers [>1.5 x IQR above 3^rd^ quartile or below 1^st^ quartile] and extreme values [>3 x IQR above 3^rd^ quartile or below 1^st^ quartile]; probability values refer to Mann-Whitney comparisons of median values).

Shrubs of the wild relative from French Guiana showed consistently higher values of Young’s modulus compared with climbing stems of the same taxon throughout development (Fig. 3 A), suggesting a distinct difference in mechanical architecture between shrubs and climbers. Values of Young’s modulus increased to a maximum for both growth forms with increasing stem diameter (c. 7000 MPa in shrubs, *c.* 5000 MPa in climbers) and then decreased somewhat in the oldest stems (Fig. 3 A). The domesticated taxon showed lower values of stem stiffness, compared with the wild relative, for the same size category for shrubs and lianas. This was particularly noticeable among shrub phenotypes of the domesticate, where median values were less than 4000 MPa. In medium-sized categories of stems of the domesticate, climbing stems were actually stiffer than the self-supporting stems (Fig. 3, B).

Tests on the bending modulus of plants from Rondônia were limited owing to an exceptionally dry season and die-back of both wild and domesticated plants.

Available material showed similar overall values of Young’s modulus for wild shrubs and climbers (M = 4180, SD = 2232, N = 36) and domesticated shrubs and climbers (M = 3250, SD = 1030, N = 14). As in French Guiana, wild climbers showed a reduction in Young’s modulus during development from approximately 6000 MPa (stems up to 10 mm diameter) down to approximately 2500 MPa (stems over 20 mm in diameter).

#### b) Maximum stress

**Figure 4:**
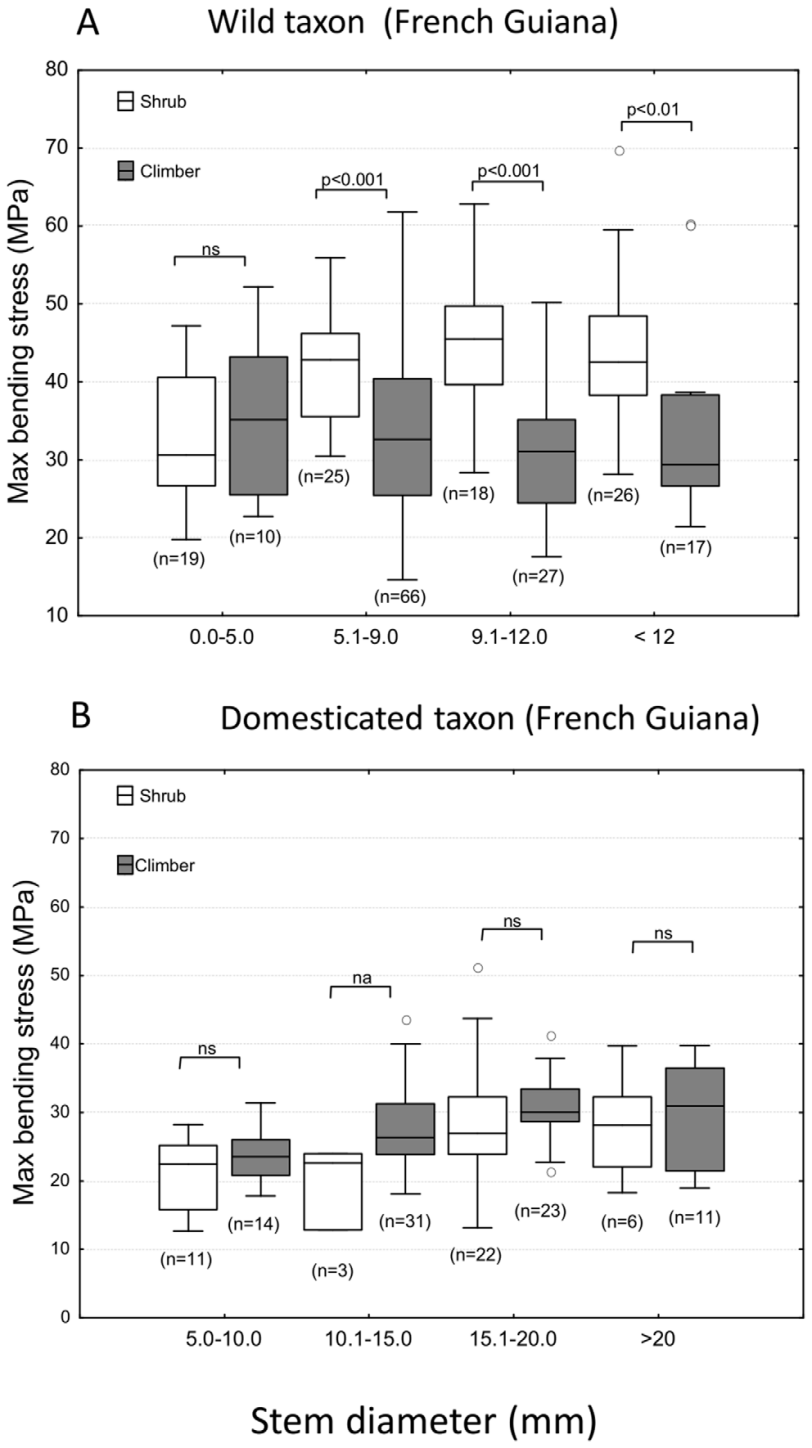
Box plots of maximum stress for shrub and climber phenotypes of wild and domesticated manioc. (A) Wild shrubs from French Guiana show consistently higher values of maximum stress compared with wild climbers. (B) There is no difference in maximum stress between shrubs and climbers in the domesticated species from French Guiana. (Graph and statistics parameters as for Fig. 3; Mann-Whitney test not performed on size class 10.1–15.0 mm because of limited sample size).

Shrubs of the wild taxon generally failed at higher maximum bending stresses than equivalent diameter stems of wild climbers - apart from the smallest diameter stems (Fig. 4, A). Shrubs and climbers of the wild taxon from French Guiana showed noticeably higher values of maximum bending stress (median values 30 – 50 MPa) compared with domesticated manioc from that region (median values 20 – 30 MPa) (Fig. 4, A, B). In domesticated manioc, maximum stress varied much less with habit and developmental stage, although there was an apparent tendency (not statistically significant in Mann-Whitney tests) for climbing stems to have marginally higher maximum stress values than self-supporting stems.

Available material from Rondônia showed mean values of maximum stress (MPa) for the wild taxon (M = 24.5, SD = 10.3, N = 40) and the domesticated taxon (M = 22.0, SD = 9.0, N = 15) that were of the same magnitude as for plants from French Guiana. There was some indication that domesticated climbers (M = 16.3, SD = 4.3, N = 6) showed mean maximum stress values that were lower than those for domesticated shrubs (M = 25, SD = 9.8, N = 9), wild shrubs (M = 23, SD = 11.1, N = 13) and wild climbers (M = 25, SD = 10.0, N = 27).

#### c) Stem failure

**Figure 5:**
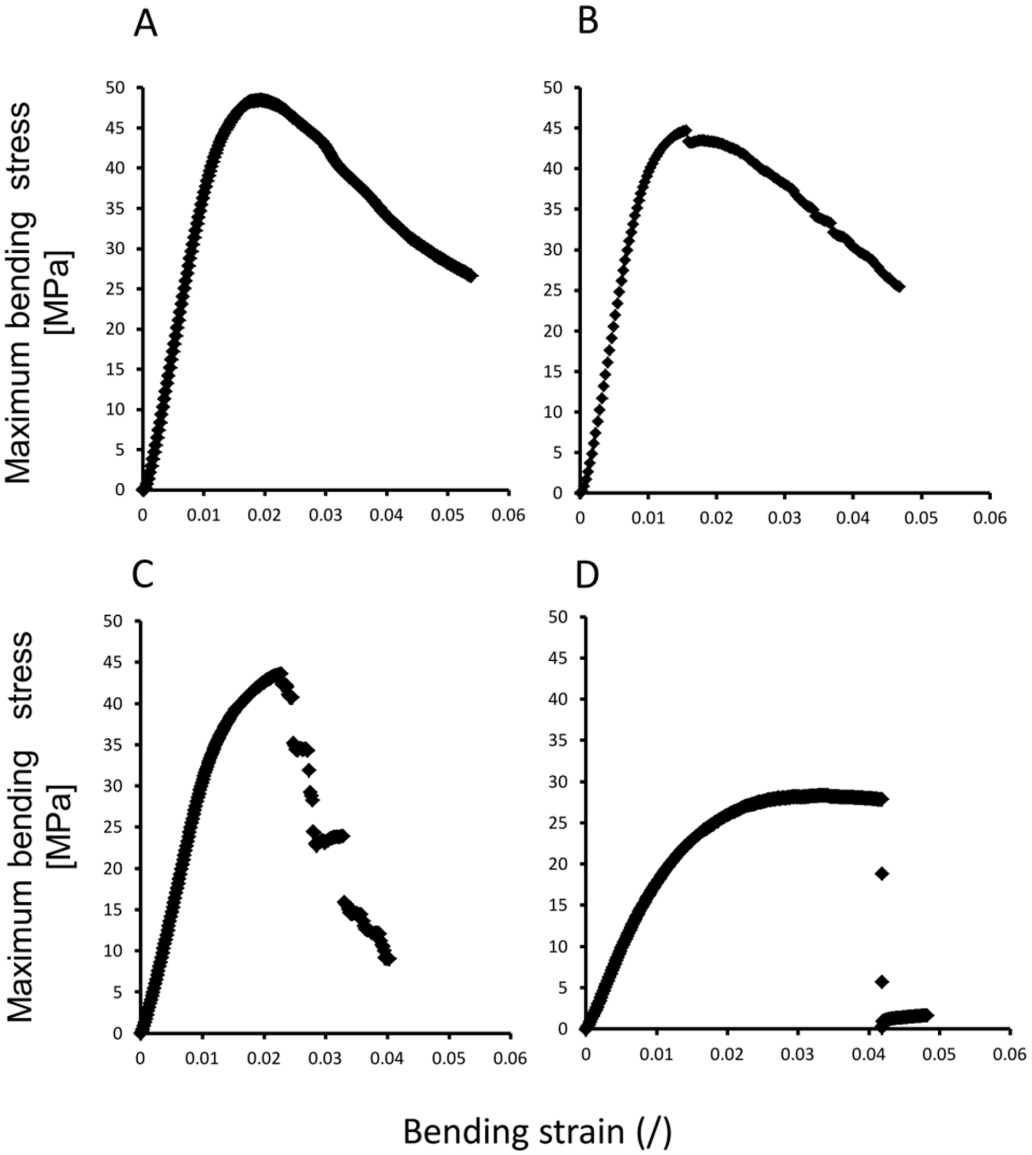
Representative stress-strain curves to failure for shrub and climber phenotypes of wild and domesticated manioc. (A) Type-1, “ductile failure”, with smooth reduction in stress not containing “stepped” reductions in stress. (B) Type-2, “sub-ductile” failure, with one or more stepped reductions in stress (each step <10% total stress) representing localized fractures of the plant stem in compression or tension. (C) Type-3, “sub-brittle” failure, with significant stepped reductions in stress (each step > 10% total stress). (D) Type-4, brittle failure, with single uninterrupted drop in measured stress. (observations in A-D are based on plants from both French Guiana and Brazil)

Values of maximum stress as measured here give an approximation of the resistance of the stem to bending forces up to failure. However, these values do not give an indication of the kind of damage or the brittleness of the plant stem. Bending tests up to failure revealed potentially interesting variations in terms of stem brittleness. As a result, different failure modes were further assessed in terms of stem brittleness by comparing stress/strain curves following yield and grouping them into the following categories of increasingly brittle behaviour (Fig. 5): Type-1 (ductile failure): smooth decline in bending stress following yield with no “steps” or sharp reductions in bending stress (Fig. 5, A). Type-2 (sub-ductile failure): Smooth decline interrupted by one or more small steps (each step <10% of maximum bending stress) indicating localized failures and probable fractures of individual fibres within the plant stem (Fig. 5 B). Type-3 (sub-brittle failure): decline interrupted by large drops in stress (each step > 10% of maximum stress) indicating substantial areas of failure and probable fractures of stem tissues (Fig. 5 C). Type-4 (brittle failure): decline interrupted by a substantial fracture of the stem with a visible fracture surface across three-quarters or more of the transverse section of the stem (Fig. 5 D).

Both shrubs and climbers of the wild taxon from French Guiana showed high frequencies of ductile or sub-ductile failure and few cases of brittle failure (Table 1). In contrast, both shrubs and climbers of domesticated manioc showed high frequencies of brittle failure and low frequencies of smooth or stepped curves (Table 1). Interestingly, type of failure did not differ much between shrubs and lianas of the wild taxon (chi^2^  =  4.98, d. f.  = 3, p<0.17) nor between shrubs and climbers (chi^2^  =  7.33, d. f.  = 3, p<0.06) of the domesticated taxon.

**Table 1:** Types of failure as measured in 3-point bending tests in stems of wild taxa in French Guiana and in Rondônia, Brazil and domesticated manioc (*Manihot esculenta* subsp. *esculenta*) from French Guiana and Brazil (results of chi^2^ tests with *P*-values comparing wild and domesticated taxa).

		Type of failure (%)					
Region	Shrub/climber	ductile	sub-ductile	sub-brittle	brittle	*n*	chi^2^
F. Guiana	Wild shrub	60	26	10	4	89	*P*<0.001
F. Guiana	Domesticated shrub	3	3	16	78	37	
F. Guiana	Wild climber	54	35	10	2	121	*P*<0.001
F. Guiana	Domesticated climber	0	1	18	81	79	
Rondônia	Wild shrubs & climbers	68	25	5	3	40	*P*<0.001
Rondônia	Domesticated shrubs & climbers	0	0	20	80	15	

See text for data on shrubs and climbers from the limited data set from Brazil.

Though limited sampling precluded a comparison of shrubs and climbers between the two taxa in Brazil, failure tests showed a similar overall pattern, with the domesticated taxon showing much more frequent brittle failures than the wild taxon (Table 1).

### Anatomy and mechanics of stems of wild and domesticated manioc

#### a) Stem and wood development

**Figure 6:**
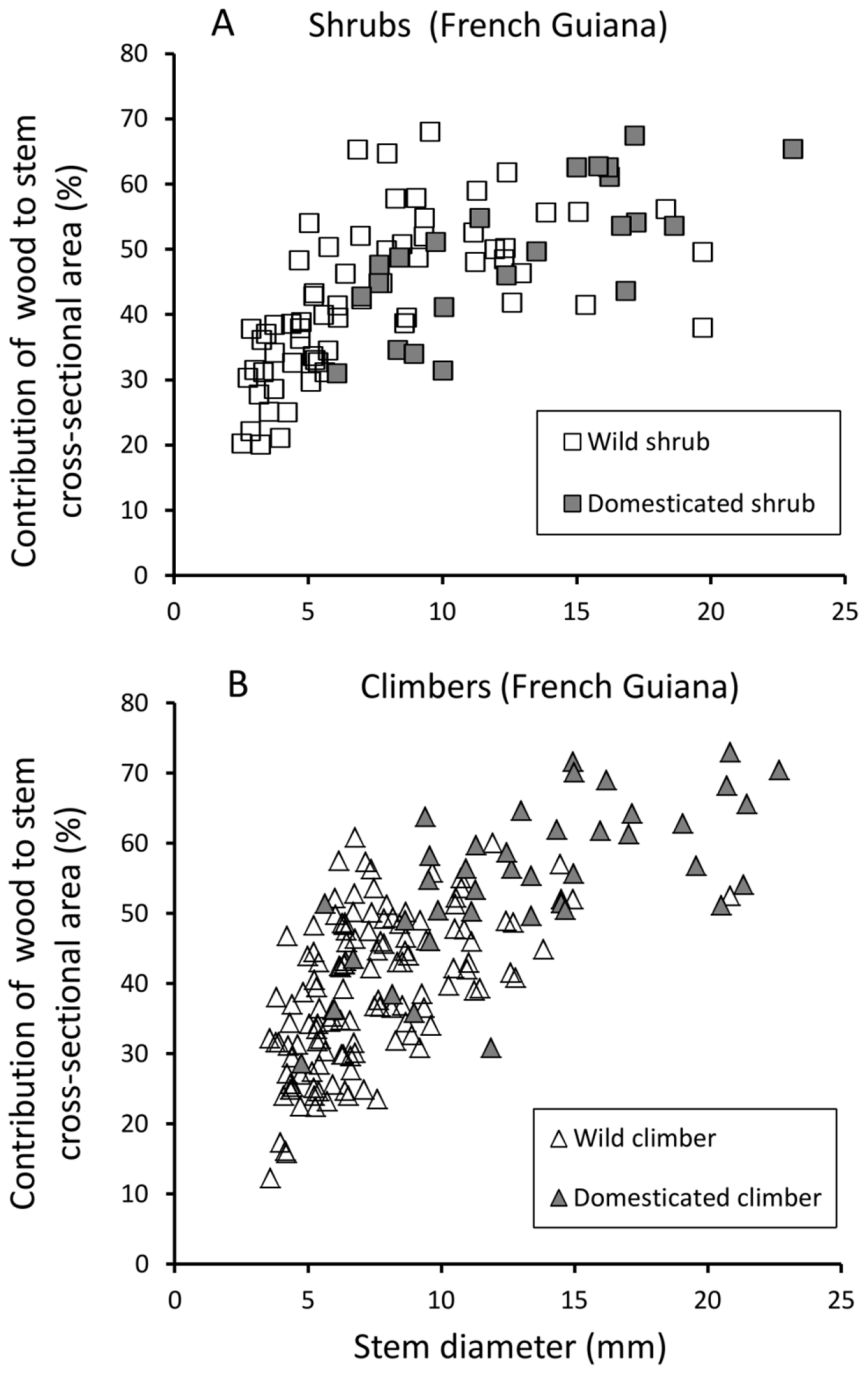
Cross sectional area of wood (%) against stem diameter (mm) of shrub and climber phenotypes. (A) Shrub phenotypes of wild and domesticated taxa from French Guiana develop similar amounts of wood in stems over 5 mm in diameter. The wild taxon develops finely tapered distal twigs with small wood cylinders which are absent in shrub phenotypes of the domesticate and only rarely present in climber phenotypes of the domesticate. (B) Climber phenotypes of the domesticate from French Guiana tend to produce thicker axes among older stems than in the wild taxon.

Both wild and domesticated taxa in French Guiana showed a similar overall development in terms of stem diameter and wood production, with stems varying from less than 5 mm in diameter at the apex to over 20 mm at the base in both shrubs and climbers. Both wild and domesticated taxa showed a similar overall production of wood varying from approximately 20–70% of the stem cross-sectional area (Fig. 6 A, B). Both taxa therefore attained a wood-dominated stem organisation relatively early in ontogeny. In both French Guiana and Brazil, shrubs and climbers of the wild taxon produced more finely tapered and narrow distal twigs compared with the domesticate, which had broader distal-most leading stems and branches (Fig. 6). In French Guiana, climbing phenotypes of domesticated manioc also produced wider basal stems than did wild climbers. This overall difference between wild and domesticate in stem diameter and tapering was also noticeable in Brazil.

#### b) Influence of wood cylinder geometry on Young’s modulus (stem stiffness) and maximum bending stress (stem strength)

**Figure 7:**
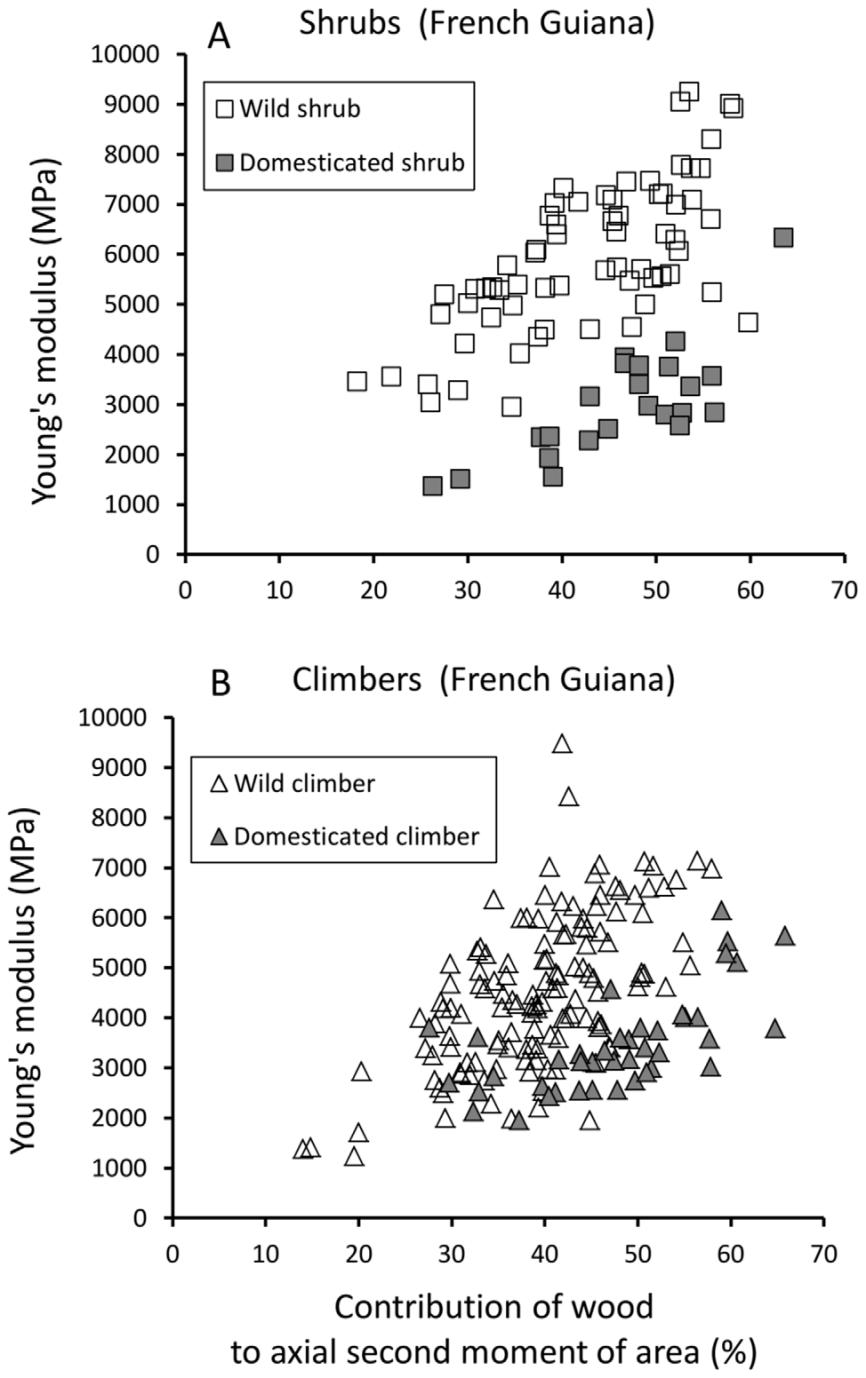
Stiffness (MPa) against proportion of wood cylinder to second moment of area of the stem. (A) Shrub phenotypes of the wild taxon show significantly higher stem stiffness for a given contribution of wood cylinder to second moment of area compared with shrubs of the domesticate. (B) A similar, but less marked difference is seen between climber phenotypes of the wild and domesticated taxa.

In both wild and domesticated taxa from French Guiana, Young’s modulus was positively correlated with the relative proportion and position of wood within the stem cross-section—expressed here as the percentage contribution of the wood cylinder to the second moment of area of the entire stem (Fig. 7 A, B, Table 2). Wild shrubs and climbers showed similar ranges of wood proportions during development as their domesticated counterparts (Fig. 7 A, B). However, both wild shrubs and climbers showed significantly higher moduli for a given wood proportion than their domestic counterparts.

**Table 2:** Wild and domesticated manioc from French Guiana: Spearman rank correlation coefficients (R_s_) of wood cylinder geometry (% contribution of wood cylinder to axial second moment of area of stem) with (i) Young’s modulus (bending) and (ii) maximum bending stress.

Wild and domesticated shrubs & climbers	R_s_	*P*-value
**(i) Young’s modulus (bending)**		
Wild shrub (n = 66)	0.6767	< 0.0001
Domesticated shrub (n = 23)	0.6719	< 0.001
Wild climber (n = 133)	0.5826	< 0.0001
Domesticated climber (n = 41)	0.6648	< 0.0001
**(ii) Maximum bending stress**		
Wild shrub (n = 70)	–0.172	0.1555
Domesticated shrub (n = 22)	0.6524	< 0.01
Wild climber (n = 82)	0.0006	0.9954
Domesticated climber (n = 38)	0.1413	0.3976

**Figure 8:**
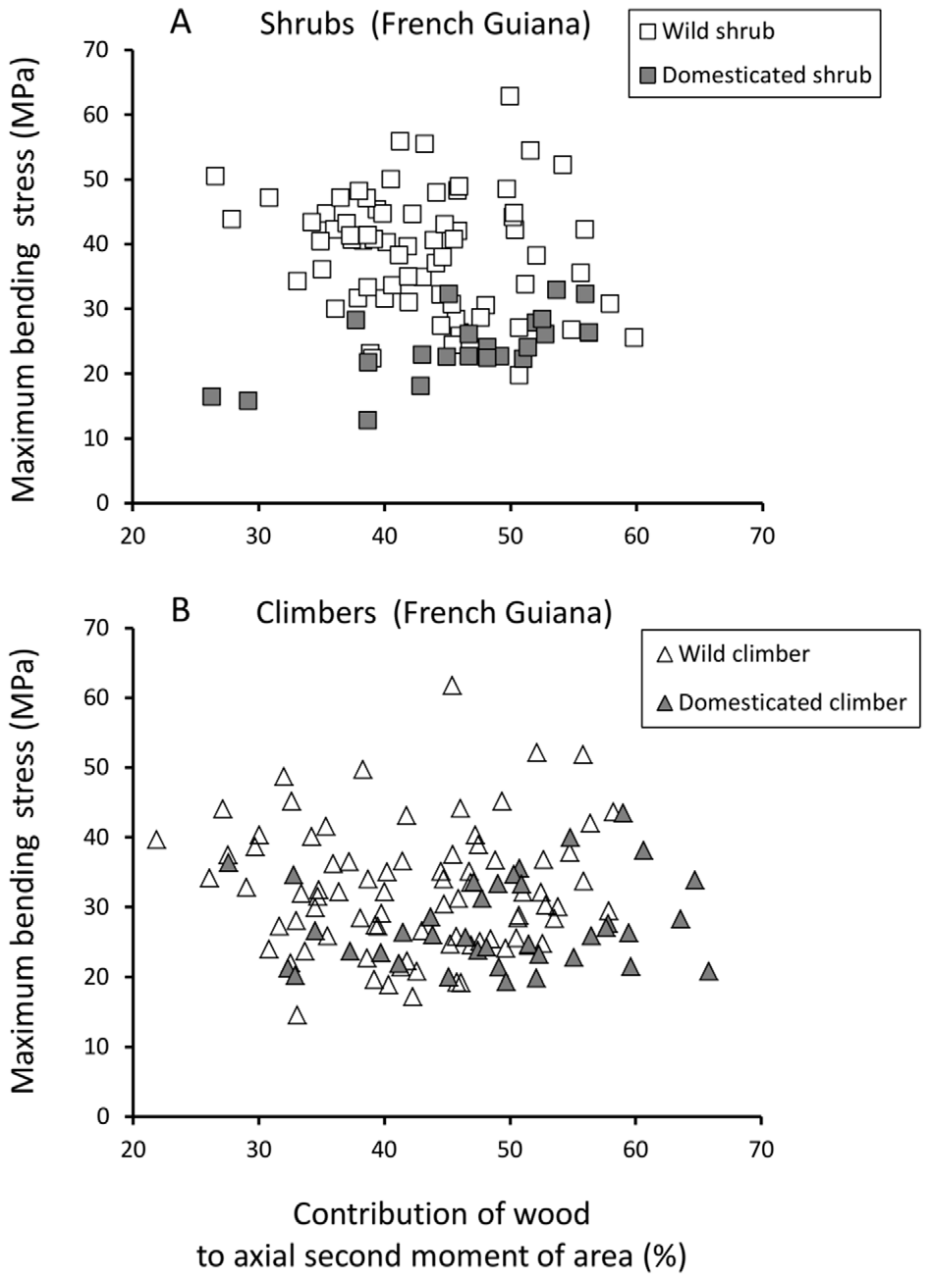
Maximum bending stress (MPa) against proportion of wood to axial second moment of area of the stem. (A) Shrub phenotypes of the wild taxon show higher values of maximum stress compared with shrub phenotypes of the domesticate. (B) Climber phenotypes of wild and domesticated taxa show little difference in maximum bending stress.

Apart from a weak positive correlation in domesticated shrubs (Table 2), Spearman rank correlations indicated that the contribution of the wood fraction to the second moment of area of the stem was not correlated with maximum stress (Fig. 8 A, B).

For a given degree of wood contribution in wild and domesticated shrubs there was generally less overlap in the scatter plot in terms of Young’s modulus (Fig. 7 A) than was observed in terms of maximum stress (Fig. 8 A). Wild and domesticated climbers, however, showed more overlap in terms of both Young’s modulus and maximum stress for a given wood contribution.

#### c) Influence of secondary xylem wall fraction on stem stiffness, strength and brittleness

Cross-sectional areas of walls and lumens of lignified xylem elements of the secondary xylem–the principal stiffening, strengthening tissues of the stem—differed significantly between wild and domesticated taxa for both growth forms (Table 3). Lignified fibre cells of wild and domesticated shrubs had the same overall cross-sectional area (wall and lumen) (Table 3) but wild-type shrubs possessed larger proportions of wall material (*c.* 15%) and smaller lumens than domesticated shrubs. A similar difference was observed between wild and domesticated climbers; in addition, domesticated climbers developed larger cells but with thinner cell walls than wild climbers (Table 3).

**Table 3:** Xylem tissue traits as measured in transverse sections (means ± s. d.) with *P*-values of Mann–Whitney tests comparing medians between shrub phenotypes of wild and domesticated manioc and between climber phenotypes of these two taxa in French Guiana.

Shrubs				Climbers		
Term	Wild (n = 27)	Domesticated (n = 31)	*P*-value	Wild (n = 26)	Domesticated (n = 24)	*P*-value
Cell wall thickness (µm)	2.43±0.46	1.58±0.42	< 0.0001	2.14±0.49	1.71±0.30	< 0.001
Cell total area (µm^2^)	241.5±46.1	264.0±56.2	0.21	242.4±47.1	305.2±58.0	< 0.001
Cell lumen area (µm^2^)	127.1±34.5	180.2±50.6	< 0.0001	139.9±41.8	207.4±55.8	< 0.0001
Cell wall area (µm^2^)	114.4±24.4	83.9±22.6	< 0.0001	102.47±22.8	97.9±18.0	0.21
Cell wall area %	47.8±8.0	32.2±7.7	< 0.0001	42.9±8.9	32.8±6.7	< 0.001

**Figure 9:**
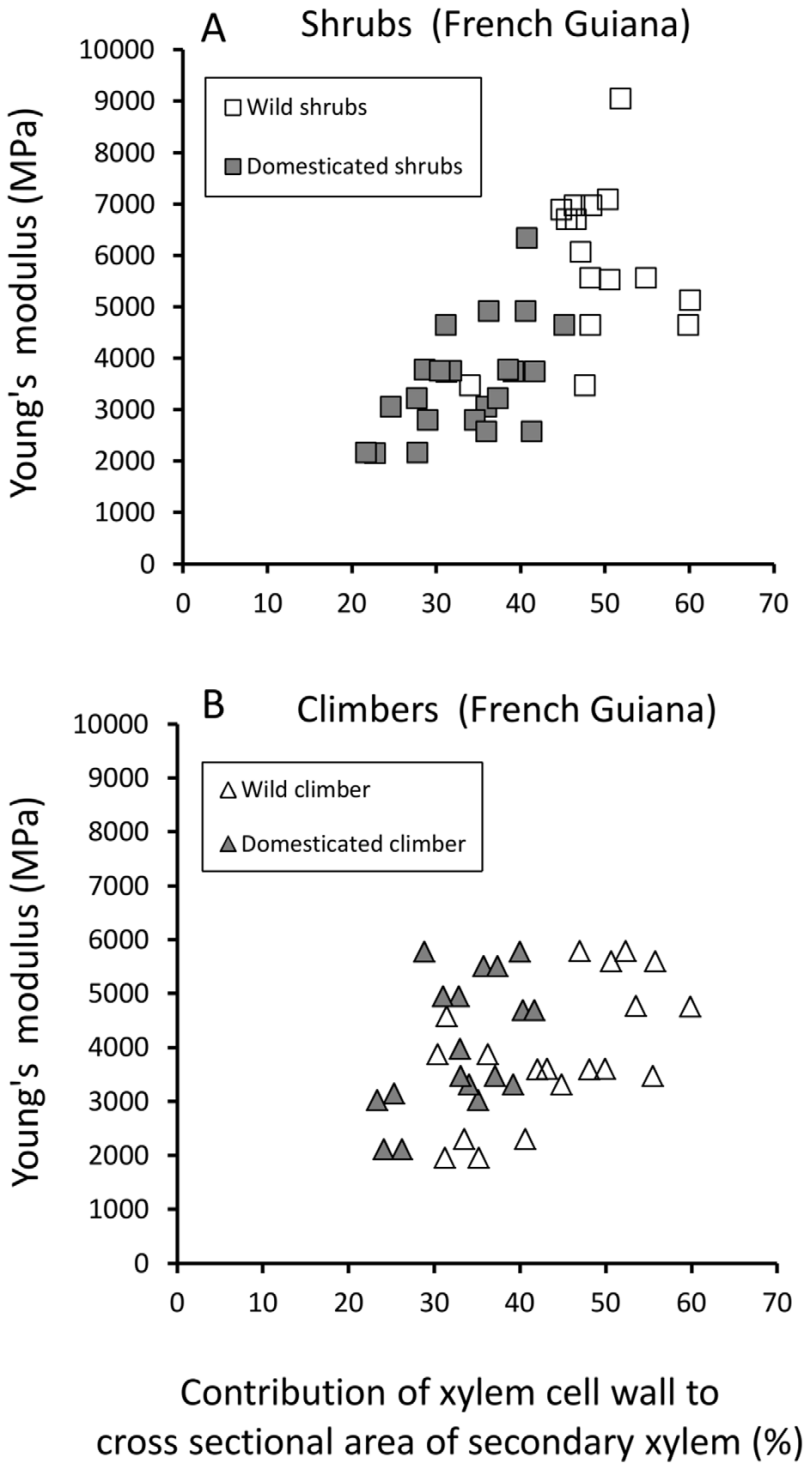
Stiffness (MPa) against xylem cell wall cross-sectional contribution to xylem tissue. (A) Shrub phenotypes of the wild and domesticated taxa. (B) Climber phenotypes of the wild and domesticated taxa.

**Figure 10:**
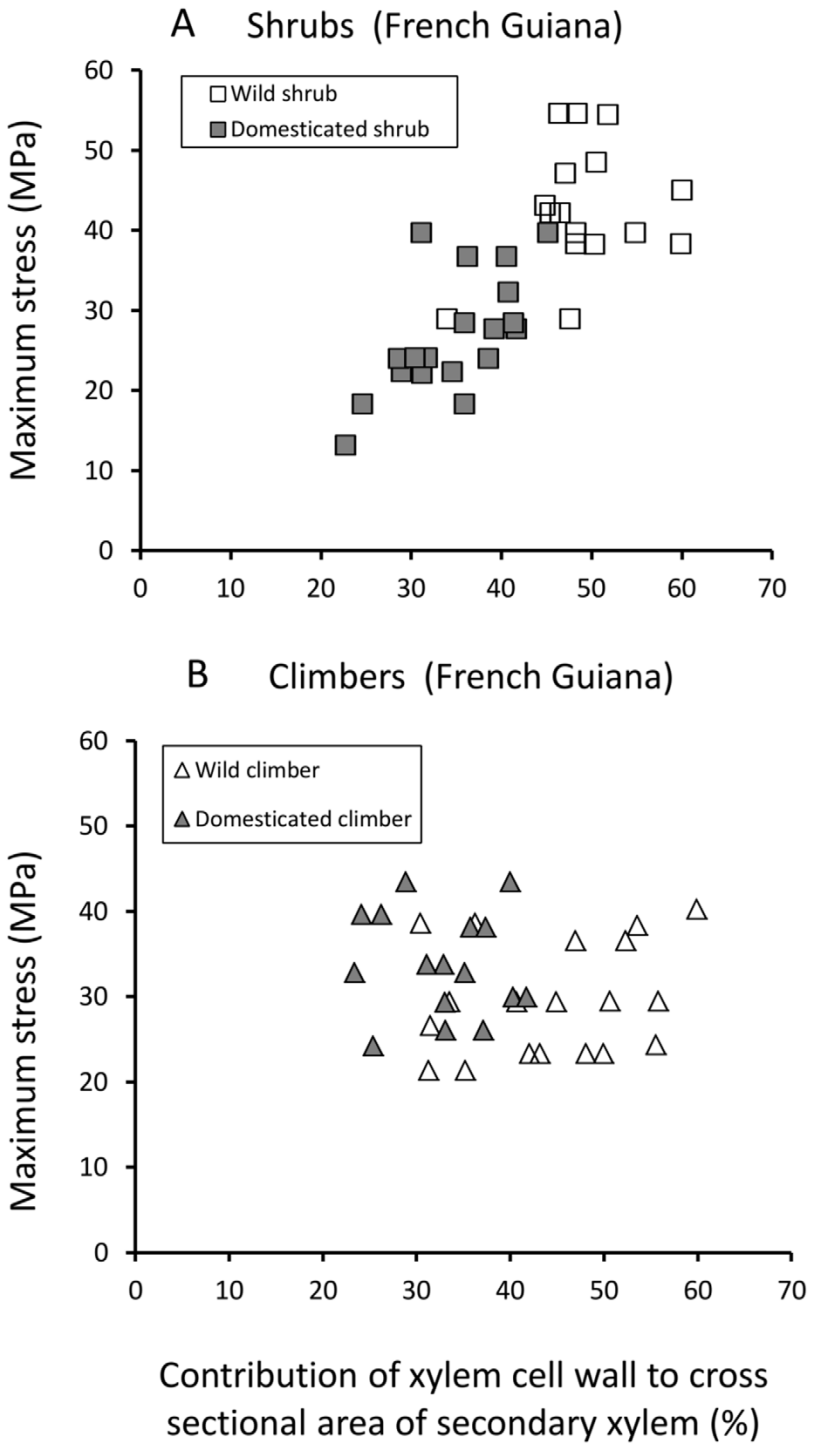
Maximum bending stress against contribution to xylem tissue. (A) Shrub phenotypes of the wild and domesticated taxa. (B) Climber phenotypes of the wild and domesticated taxa.

Scatter plots of xylem cell wall fraction against Young’s modulus (Fig. 9 A) and against maximum stress (Fig. 10 A) indicate that shrubs with thicker-walled xylem elements are stiffer and more resistant to failure than shrubs with thinner-walled xylem tissue. Among climbers, the pattern of fibre density and mechanical properties between wild and domesticated taxa differed from that of shrubs; although wild climbers generally were found to have thicker cell walls there was no discernible difference in stiffness (Fig. 9 B) or maximum stress (Fig. 10 B) compared with domesticated climbers.

**Figure 11:**
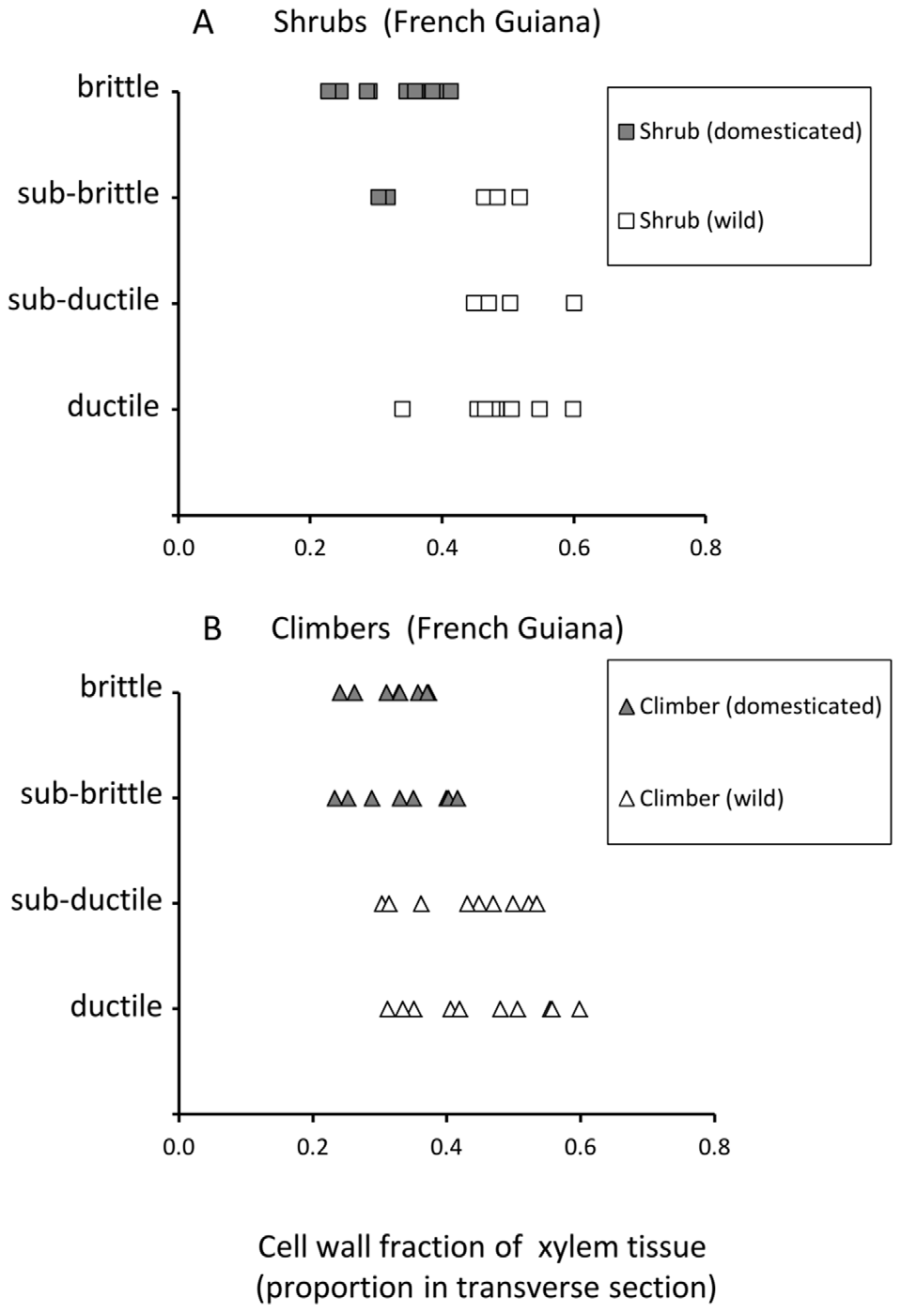
Cell wall fraction of xylem tissues against category of stem failure. (A) Shrub phenotypes of the wild and domesticated taxa. (B) Climber phenotypes of the wild and domesticated taxa.

In shrubs of the domesticated taxon, xylem wall fraction was linked with stem brittleness (Fig. 11 A). Domesticated shrubs with xylem wall fractions less than 0.4 (with the exception of two stems) all showed either brittle or sub-brittle failure. Wild shrubs all showed values of xylem wall fraction above 0.4 (with the exception of one stem) and most stems showed ductile or sub-ductile failure with only three showing sub-brittle failure.

Climbing phenotypes of domesticated plants showed the same degree of xylem wall fraction as domesticated shrubs (Fig. 11 B) but showed a higher proportion of sub-brittle failure. In wild climbers, there was a noticeable decrease in cell wall fraction and approximately half of the tested stems showed the same cell wall thickness as the domesticated climbers. Despite this similarity in wall thickness, wild climbers showed only sub-ductile and ductile failure properties.

#### d) Vessel size and density

Wild shrubs had significantly higher numbers of vessels per mm^2^ in transverse sections compared with domesticated shrubs (Table 4); a similar pattern was seen among wild and domesticated climbers (Table 4). Individual vessel lumen areas were not significantly different between corresponding growth forms of wild and domesticate. Wild shrubs and climbers therefore developed greater vessel lumen areas per area of wood than their domesticated counterparts as a result of increased vessel density rather than producing wider vessels.

**Table 4:** Vessel size and density traits (means ± s. d.) with *P*-values of Mann–Whitney tests comparing medians between shrub phenotypes of the wild and domesticated manioc and between climber phenotypes of these two taxa.

Shrubs				Climbers		
Term	Wild (n = 39)	Domesticated (n = 31)	*P*-value	Wild (n = 31)	Domesticated (n = 23)	*P*-value
Vessels per mm^2^	14.0±3.47	9.61±4.27	< 0.0001	17.8±4.35	9.62±4.34	< 0.0001
Vessel lumen area (µm^2^)	6705±2203	7790±3218	0.23	6609±2145	8128±3304	0.087
Vessel lumen area/wood area	0.089±0.023	0.065±0.016	< 0.0001	0.114±0.033	0.069±0.027	< 0.0001
Vessel lumen area of wood %	8.91±2.3	6.483±1.6	< 0.0001	11.35±3.35	6.95±2.65	< 0.0001

#### e) Microfibril angle

**Figure 12:**
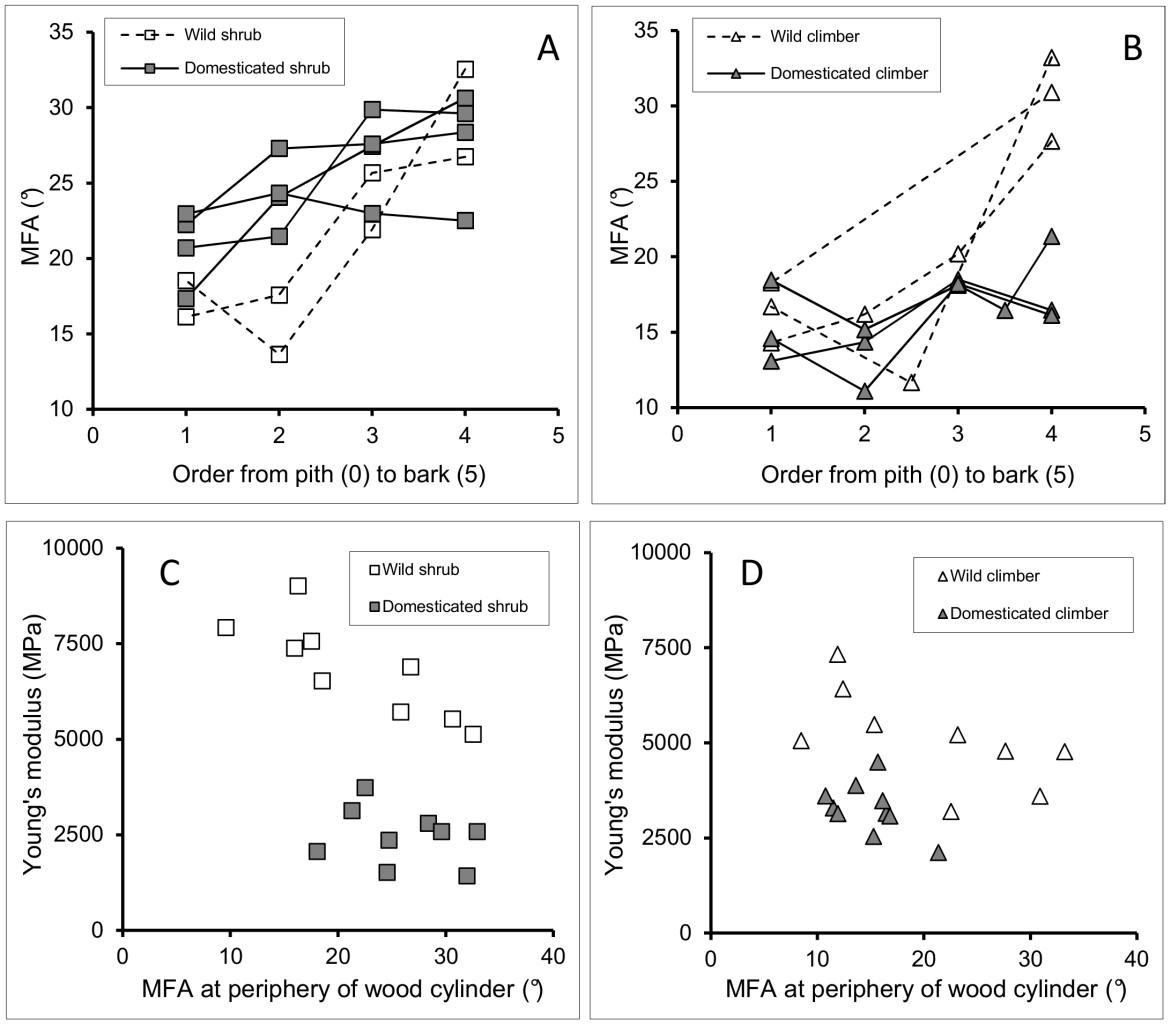
Microfibril angle for shrub and climber phenotypes of wild and domesticated manioc in French Guiana. (A) MFA at relative distances within wood cylinder from pith to bark for wild and domesticated shrubs. (B) MFA at relative distance within wood cylinder from pith to bark for shrubs and climbers of the domesticate. (C) Young’s modulus against MFA at periphery of wood cylinder for shrub phenotypes of the wild and the domesticated taxon. Each symbol represents a segment of stem in the range of 0.3 to 2.5 m above ground level. (D) Young’s modulus against MFA at periphery of wood cylinder for shrubs and climbers of the domesticate. Each symbol represents a segment of stem in the range of 0.3 to 4.0 m above ground level.

In both wild and domesticated shrubs and climbers, MFA increased from the inner to the outer part of the wood cylinder (Fig. 12 A, B). However, the degree of change was always less in the domesticate and greater in the wild taxon. In shrub phenotypes, MFA was lower in the wild taxon (16–19°) than in the domesticated taxon (20–23°) in the early stage of growth (near the pith) whereas this difference disappeared in the later-formed tissues (Fig. 12 A). In contrast, climber phenotypes exhibited little difference between wild and domesticate near the pith but the difference became significant in the peripheral tissues, in which MFA was much greater in the wild taxon (27–33°) than in the domesticate (16–21°) (Fig. 12 B). In the wild taxon, there was no discernible difference in MFA between climbers and shrubs (Fig. 12 A, B) but in domesticated plants, climbers had consistently lower MFA (c. 10–20°) than shrubs (c 20–30°). In both wild and domesticated plants, Young’s modulus of wood samples generally decreased with increasing MFA (Fig. 12 C, D). However, the wild taxon showed consistently higher values of Young’s modulus than the domesticate for a given microfibril angle (Fig. 12 C, D), for both shrub and climber phenotypes. Climber phenotypes of the domesticate showed consistently lower MFA than did shrubs, which had marginally higher values of elastic modulus than did the climbers.

## Discussion

### Retention of growth form plasticity after domestication

Phenotypic plasticity refers to the capacity of a genotype to express different phenotypes as a function of the environment [59]. Because of the near-perfect correspondence between phenotype (shrub or climber) and environment (savannah or forest) in populations of the wild taxon, it can be supposed that the growth form and corresponding mechanical architecture were modified directly in response to the environment. If the differences were due to genetic variation, this would imply a perfect correspondence between genotype and environment, and this seems highly unlikely. Furthermore, individuals in transitional environments had begun to show a transition in phenotypes; for example, in French Guiana at the edges of forest patches we found self-supporting individuals of the wild taxon that had developed long unstable axes typical of early phases of climbing growth. The differences observed between self-supporting and climbing phenotypes were thus most likely due to developmental plasticity of the stem in response to environment and not an effect of genetic variation. Nevertheless, experiments using the same genotype under different environmental conditions would be required to exclude strictly a genetic contribution to the observed differences. An experimental approach would also clarify the mechanisms by which environment affects phenotype, for example whether the phenotypic response to develop as a shrub or as a climber is driven by variations in light intensity or by thigmomorphogenetic reactions such as growth responses to mechanical stimuli in the local environment.

Both the ancestral wild taxon and the domesticate, from both areas of Amazonia, show a high degree of developmental plasticity where plants grow as shrubs in open habitats and as lianas in closed conditions. Results from comparison of wild and domesticate in French Guiana indicate that these different growth forms develop significantly different mechanical architectures in terms of stiffness, strength and brittleness as well as in overall size and shape. These observations are consistent with accounts of growth habit variation in manioc that have described both shrubs and climbers [26], [41], [60]. Furthermore, this kind of broad phenotypic plasticity has also been documented in another wild species of *Manihot*, *M. quinquepartita*, which develops shrubs and treelets in open habitats but truly canopy-climbing lianas in forest habitats [23] that attach by the same kind of branch-angle mechanism observed in manioc. Studies elsewhere have demonstrated a diversity of other life forms in *Manihot* ranging from short-stemmed rosette-forming shrubs to trees [45]. Domestication does not appear to have modified the tendency seen in the genus to produce shrubs or lianas depending on the local habitat. The retention of growth form plasticity in domesticated manioc differs from the kinds of change in architecture known to occur in other cases, such as maize and its wild ancestor teosinte, in which domestication strongly influenced growth form plasticity in open or closed conditions [4].

Domestication is known to reduce the degree of branching in other domesticates including millet [7], rice [8] and bean [9]. Although the amount of branching was not quantified in this study, many domesticated individuals we saw possessed thicker, less tapered stems compared to the wild plants. While some aspects of stem geometry and branching have undoubtedly been modified during the domestication of manioc [61], domestication did not involve loss of the recurved, wide-angled branches that enable attachment in climbing phenotypes and which thus contribute to the high level of growth form plasticity after domestication.

In the wild taxon from French Guiana, older stems of shrubs were significantly stiffer, showing significantly higher Young’s moduli than older stems of climbers. This is consistent with biomechanical comparisons of climbers with shrubs and trees, where older, basal parts of self-supporting growth forms are built of stiffer materials than the equivalent basal stages of non-self-supporting plants [17]. Although climbers of the wild taxon develop lower stiffness than do shrubs of this taxon, they do not develop the extremely low values of Young’s modulus typical of many twining lianas. The potential to develop shrubs and treelet-sized individuals in addition to climbing growth forms is possibly linked to a less specialized kind of climbing architecture compared with some lianas that develop highly flexible stems but not relatively large-bodied self-supporting stages of growth [22]. Furthermore, the mode of attachment via wide-angle branches, where stems and branches need to retain a degree of stiffness to maintain an attachment, is more consistent with retention of stiffer material properties rather than high flexibility [18], [23].

### Domestication reduced the stiffness and strength of stems and increased their brittleness

Despite the retention of developmental plasticity in terms of overall morphology, domestication resulted in (a) lower stiffness, (b) reduced difference in mechanical properties between shrubs and climbers, and (c) increased brittleness. Observations based on shrub and climber phenotypes of the wild and domesticated taxa from French Guiana were consistent with those from Rondônia, where stiffness of shrubs and climbers of the domesticate was generally no higher than 4000 MPa compared with up to 4000–6000 MPa in shrubs and climbers of the wild taxon. Shrubs and climbers of the domesticate from both areas were also less resistant in terms of maximum bending stress at failure compared to the wild taxon.

Stems of the domesticate were significantly more brittle than those of the wild taxon, showing consistently higher frequencies of failure types described as sub-brittle and brittle. Sub-brittle failure largely corresponded to localised, step-like fracture (either transverse fracture or fibre pull-out) from the periphery to the centre of the wood cylinder. Brittle behaviour corresponded to a rapid propagation of a fracture surface across most or all of the stem cross-section. Brittleness was prevalent in domesticated manioc from both French Guiana and Brazil, suggesting that the phenomenon is general. The results explain our observations of brittle failure of climbing stems of the domesticate and point to a structural weakness that potentially limits their survival as climbing stems under natural conditions.

We suspect that the lower stiffness and lower maximum stress of stems of the domesticate might be two of the underlying reasons why the domesticate, as we observed, did not generally develop finely tapered distal shoots as found in the wild taxa. Stems of the domesticate from French Guiana generally showed only 50% the stiffness of stems of the wild taxon for a given stage of development and this would substantially influence stem rigidity, instability and risk of buckling among very slender shoots.

### What scales of organisation and development modify mechanical architectures during domestication?

#### a) Wood cylinder geometry and secondary growth

The contribution of the wood cylinder to the second moment of area of the stem is a key mechanical attribute in woody plants. It represents the amount of wood within the stem cross-section at a given stage of development as well as its effectiveness in resisting bending forces relative to the centrally located neutral plane [52]. Anatomical observations on wild and domesticated manioc from French Guiana showed that size and position of the wood cylinder were unchanged between the two. Changes of stiffness, strength and brittleness were therefore linked to changes in properties at a finer scale of organisation.

#### b) Xylem tissue and cell wall thickness

Wood toughness can depend on a variety of factors [62] including general wood density [63], [64], cell wall thickness [65], cell wall thickening and cellulose microfibril organisation [66] as well as larger-scale tissue organisations such as wood ray organisation [67]. Shrub phenotypes of the domesticate from French Guiana developed secondary xylem cells with significantly thinner walls and larger lumens than shrubs of the wild taxon. This potentially explains the lower stiffness and strength as well as greater brittleness in shrubs of the domesticate. However, additional factors must be at work among climbers, since stiffness and strength were less well correlated with xylem wall fraction and a large proportion of climbers of the wild taxon developed equally thin walls but showed less brittle failure.

Greater toughness in climbers may result from a combination of micro-organisational features possibly related to vessel density and size, xylem parenchyma and size and orientation of ray tissue. Studies focussing on plant toughness with respect to herbivory have discussed the possibility that vessel size may play a role in wood toughness [68], where large numbers of large vessels can reduce stem toughness in scissor-cutting toughness tests. Our results suggest that the absence of brittle and sub-brittle behaviour in lianoid stems of the wild taxon might be linked to higher frequencies of vessels, which reach up to 11% of the wood cross-sectional area. Such an arrangement is considerably less than the 50% vessel area observed in lianoid stems of *Bauhinia* [68], which showed low toughness in scissors toughness tests compared with tree wood. A first-order interpretation might suggest that up to a point higher vessel frequencies might limit transverse fractures during extreme bending and shearing via crack-stopping [69], [70].

#### c) Microfibril angle

In most woody plants studied (mainly trees), microfibril angles of wood fibres change within the wood cylinder from relatively large angles (from the vertical axis of the fibre cell) at the inside of the wood cylinder to relatively small angles at the outer part of the wood cylinder [71]. These changes are known to be linked with variations from low to high stiffness [72]. This is believed to be linked with a mechanical organisation in which distal or juvenile parts of the stem need to be relatively flexible and deform easily in wind, whereas basal, stiffer supporting parts of the plant stem need to be rigid.

Microfibril angle in both the wild and the domesticated taxon in French Guiana shows an increase in MFA from the inner to the outer part of the wood cylinder. This differs from most tree species but is consistent with the mechanical strategy among climbing plants [17], [73], where early juvenile stages of growth are nearly always optimised for stiff “searching” behaviour and older stages of growth are comprised of more flexible material, once the plant has attached to its support. A recent study has also found a decrease in stiffness from the inside to outside of the stem in tropical tree species grown in closed forest conditions [74], implying that MFA can change in response to open or closed environmental conditions in tree species.

Closed habitats with greater likelihood of shade, less light and more shelter from the wind, possibly lead to the initiation of climbing modes of growth. Climbers such as the closest wild relatives of manioc that attach to vegetation via branches develop height before attaching to the surrounding vegetation. An early phase of climbing, general to many woody climbing plants, involves maximising height and span distance while minimising the physiological cost of growth, particularly in shaded environments. Both shrub and climber phenotypes of both wild and domesticated manioc develop an increase in wood microfibril angle from pith to bark, which is arguably consistent with the overall strategy and mechanical requirement of a woody climber – initially stiff properties followed by compliant stem properties.

#### d) Differences in MFA between shrub and climber phenotypes of the domesticate

In the wild taxon, there was no significant difference in microfibril angle between shrubs and climbers. However, shrub and climber phenotypes of the domesticate show different ranges of microfibril angles, with values for climbers remaining lower than those for the shrubs. This might explain why wood from climbers of the domesticate is marginally stiffer than that of shrubs of the same taxon. However, the effects on mechanical properties are difficult to interpret directly, since domestication has also involved such a significant change in xylem cell-wall thickness as well. In the domesticate, allocation of resources has possibly been modified towards storage of mobile carbohydrates in the underground root system of manioc. Since MFA at the outer part of the wood cylinder is correlated negatively with Young’s modulus in both shrubs and climbers, retention of low microfibril angles might represent a mechanism for maintaining stem stiffness if allocation of resources towards cell wall thickness is limited.

What mechanisms and selective pressures drove changes in the biomechanical properties of manioc stems?

#### a) Relaxed selective pressures led to reduction of biomechanical traits conferring success as a liana

The plant’s ecological niche has been altered by domestication. The wild ancestor has a high level of growth form plasticity involving the ability to grow as shrubs or lianas. This plasticity contributes to its success in a mosaic of different habitats and periodic disturbances [23], [47]. Whereas the wild ancestor grows as a self-supporting shrub or as a liana, depending upon successional stage, domesticated manioc is managed as a shrub in open environments. This change in niche should have led to relaxed selective pressures on biomechanical properties such as flexibility and toughness that contribute to success as a liana. The brittleness of stems of domesticated manioc is probably the most important factor limiting the persistence of climbing phenotypes in abandoned swidden plots. Thus, even in the rare cases when plants are not uprooted before a field goes into fallow, any remaining plants are unlikely to survive until the plot is farmed again. There is thus no selection pressure that would maintain, in domesticated manioc, adaptations to climbing in fallow vegetation.

#### b) Biomechanical traits changed as a pleiotropic consequence of selection on other traits

Many of the differences in stems of domesticated manioc and its wild relatives seem to reflect a recurrent pattern under domestication, namely a shift from a strategy that emphasizes resource conservation to one that places a greater emphasis on resource acquisition [11], for example, by reduced investment in structural carbohydrates of cell walls and increased investment in non-structural carbohydrates. Such changes, driven by other forces, will affect stem biomechanical properties such as the degree of suppleness or brittleness.

**Figure 13:**
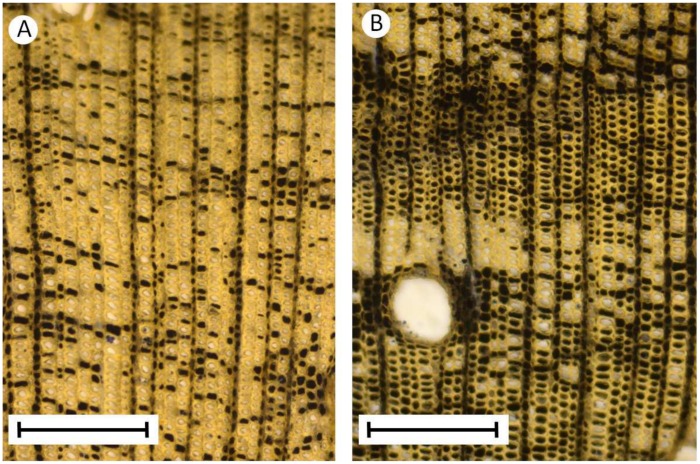
Transverse sections of wood cylinders showing different starch grain densities from French Guiana. (A) Wild shrub (B) domesticated shrub Sections were made at a thickness of 60 µm and stained with 1% iodine solution in 90% ethanol for 5 minutes; scale bar  =  250 µm).

Changes in biomechanical properties could also be a consequence of selection on chemical and anatomical composition of manioc stems driven by selection acting on a function newly acquired under domestication, i.e. to serve as clonal propagules. Selection acting on this new function should have favoured stems with increased content of energy reserves and increased meristematic potential to form adventitious roots and new shoots. These new adaptations involve anatomical modifications that affect stem mechanical properties. In fact, stems of domesticated manioc do contain much greater concentrations of starch than those of its wild relatives (Fig. 13, compare A and B). This difference might simply be one reflection of an overall tendency, in all parts of the plant, towards increased allocation to starch production in domesticated manioc, accompanied by reduced allocation to structural carbohydrates. However, selection under domestication may also have acted directly to increase starch storage in stems, independently of selection for increased starch storage in tuberous roots. As the principal energy reserve, starch plays a decisive role in the quality of stems of domesticated manioc as propagules for vegetative propagation. Human selection could have favoured modifications in the structure of wood that led to increased accumulation of starch.

Preliminary observations suggest that wood cylinders of both the wild and domesticated taxa develop relatively large amounts of xylem parenchyma containing starch grains, which appear to be more densely distributed in stems of the domesticate. On-going studies will elucidate what developmental changes occur during cambial development in (1) the transition from juvenile to mature growth, (2) stem development as shrubs or lianas and (3) changes at the cellular level of cambial derivatives of fibre and parenchyma formation during domestication. Whatever the exact modifications of wood formation during domestication, a change in biomass allocation was probably pivotal in lowering xylem cell wall thickness, loss of mechanical specialization between shrubs and climbers, reduced stiffness and strength and increased brittleness in stems of the domesticate.

Another distinctive trait of stems of domesticated manioc in comparison to its wild relatives is the much more pronounced volume of parenchymatous tissue either side of each leaf scar at the nodes. It is from these zones that adventitious roots are produced from stem cuttings of domesticated manioc, and the greater size of these zones has been considered as an adaptation to facilitate clonal propagation [61].

#### c) Human selection directly favored more brittle stems in domesticated manioc

Lacking the metal tools that farmers everywhere today use to prepare stem cuttings of manioc, the earliest cultivators of manioc may have prepared propagules by breaking stems, selectively propagating plants whose stems broke most readily and cleanly. Although the hypothesis is difficult to test, selection by early farmers might thus have directly favoured increased brittleness of stems during the initial domestication of manioc. Interestingly, when stems of manioc rupture—and their abrupt and total rupture is one of the strongest contrasts in mechanical properties they present in comparison to the wild relatives—they usually rupture at the enlarged nodal parenchymatous zones that appear to facilitate production of adventitious roots. These zones might thus contribute to the brittleness observed in domesticated stems.

#### d) Physiological age of propagules

Selection of cuttings from adult plants and their subsequent cultivation as independent plants can influence wood characteristics because growth onset in these vegetative propagules occurs at a different physiological age from that of plants grown from seed. Such processes can influence both wood density and microfibril angle [75]. Selection by farmers of relatively mature stem segments for use as propagules [29] might therefore “fix” the physiological and cambial age of ramets of domesticated manioc and thus influence their mechanical properties. Shrub phenotypes of the domesticate that we observed showed early-formed wood MFA that were higher than early wood MFA from shrub and climber phenotypes of the wild taxon, though a larger sampling would be necessary to substantiate this.

The higher microfibril angles observed in early-formed wood in shrubs of the domesticate possibly reflect the older physiological age and ultrastructural properties present in older stems used for vegetative propagation. It might be expected that early-formed wood in the domesticate might be predisposed to show “mature” traits if the domestication and vegetative propagation process “fixed” mature wood traits.

Early-formed wood MFA of domesticated plants growing as climbers, however, showed comparable values as early growth from the wild taxon. This suggests that growth under a modified physiological age following domestication might be overridden by environmental cues and high phenotypic plasticity. In this case, growth in shaded, understory conditions as a climber overrode physiologically fixed wood properties and phenotypes developed early wood with low MFA angle characteristic of wild relatives.

### Evolution and “instant domestication”

Ever since Darwin, investigations of domesticated plants and animals have made key contributions to the study of evolution; for recent examples, see [76]–[81].

Among plants, however, most work on evolution under domestication has focused on seed-propagated plants, particularly cereals and legumes. The evolution of crop plants propagated clonally by farmers has received relatively little attention. This has been due in part to the perception that domestication under clonal propagation is a simpler process, which could in some cases even occur “instantaneously” with the vegetative multiplication of wild plants with desired characteristics. However, recent work has shown that mixed clonal/sexual reproductive systems allow the accumulation under domestication of numerous independent traits via repeated recombination/selection cycles, as in seed-propagated crop plants [10], [11]. Our study adds to the list of sometimes surprising [47] traits that have been affected by domestication in *Manihot*.

Our results indicate that in *Manihot* domestication had far-reaching effects on the mechanical architecture of the stem and its likely resistance to mechanical perturbation and brittleness. We found that, although the domesticate retained a level of phenotypic plasticity similar to that of the wild ancestor, in terms of being able to produce shrub or liana growth forms depending on environmental conditions, domestication produced changes that had profound consequences for the ecological success of liana phenotypes of the domesticate. These changes affected relatively small-scale features of the plant stem, particularly wall thickness of lignified tissue, and microfibril angle. As a consequence of these changes, long climbing stems of domesticated manioc are vulnerable to brittle failure. They have thus lost a key adaptive trait that characterizes most woody climbing plants. The management of manioc by farmers strictly as a shrub in open environments thus ultimately facilitated the loss of some adaptations of the wild ancestor to life as *either* a shrub or a liana, in response to changing environmental conditions such as dynamic forest/savannah ecotones.
